# Origins and Evolution of the Etruscans’ mtDNA

**DOI:** 10.1371/journal.pone.0055519

**Published:** 2013-02-06

**Authors:** Silvia Ghirotto, Francesca Tassi, Erica Fumagalli, Vincenza Colonna, Anna Sandionigi, Martina Lari, Stefania Vai, Emmanuele Petiti, Giorgio Corti, Ermanno Rizzi, Gianluca De Bellis, David Caramelli, Guido Barbujani

**Affiliations:** 1 Department of Biology and Evolution, University of Ferrara, Ferrara, Italy; 2 Department of Biotechnologies and Biosciences, University of Milano-Bicocca, Milan, Italy; 3 Institute of Genetics e Biophysics “Adriano Buzzati-Traverso”, National Research Council, Naples, Italy; 4 Department of Evolutionary Biology, University of Florence, Florence, Italy; 5 Institute for Biomedical Technologies, National Research Council, Segrate, Milan, Italy; University of Wisconsin, United States of America

## Abstract

The Etruscan culture is documented in Etruria, Central Italy, from the 8^th^ to the 1^st^ century BC. For more than 2,000 years there has been disagreement on the Etruscans’ biological origins, whether local or in Anatolia. Genetic affinities with both Tuscan and Anatolian populations have been reported, but so far all attempts have failed to fit the Etruscans’ and modern populations in the same genealogy. We extracted and typed the hypervariable region of mitochondrial DNA of 14 individuals buried in two Etruscan necropoleis, analyzing them along with other Etruscan and Medieval samples, and 4,910 contemporary individuals from the Mediterranean basin. Comparing ancient (30 Etruscans, 27 Medieval individuals) and modern DNA sequences (370 Tuscans), with the results of millions of computer simulations, we show that the Etruscans can be considered ancestral, with a high degree of confidence, to the current inhabitants of Casentino and Volterra, but not to the general contemporary population of the former Etruscan homeland. By further considering two Anatolian samples (35 and 123 individuals) we could estimate that the genetic links between Tuscany and Anatolia date back to at least 5,000 years ago, strongly suggesting that the Etruscan culture developed locally, and not as an immediate consequence of immigration from the Eastern Mediterranean shores.

## Introduction

The Etruscan culture is documented in Central Italy (current Tuscany and Northern Latium, formerly known as Etruria) between the 8^th^ and the 1^st^ century BC. Questions about the Etruscans’ origins and fate have been around for millennia. Herodotus and Livy regarded them as immigrants, respectively from Lydia, i.e. Western Anatolia, or from North of the Alps, whereas for Dionysius of Halicarnassus they were an autochthonous population [1]. Previous DNA studies, far from settling the issue, have raised further questions. The Etruscans’ mitochondrial DNAs (mtDNAs) appear similar, but seldom identical, to those currently observed in Tuscany [2], [3]. Assuming reasonable effects of genetic drift and mutation, these levels of resemblance proved incompatible with the notion that modern Tuscans are descended from Etruscan ancestors [4], [5]. Explanations for this result include the (extreme) possibility that the Etruscans became extinct, but also that their modern descendants are few and geographically dispersed, or that the ancient sample studied represents a small social elite rather than the entire population [4]. As for the Etruscans’ origins, ancient DNA is of little use, because pre-Etruscan dwellers of Central Italy, of the Villanovan culture, cremated their dead [1], and hence their genetic features are unknown. DNAs from modern humans and cattle in Tuscany show affinities with Near Eastern DNAs, which was interpreted as supporting Herodotus’ narrative [2], [6], but in these studies modern Tuscans were assumed to be descended from Etruscan ancestors, in contrast with ancient DNA evidence [5]. The claim that systematic errors in the Etruscan DNA sequences led to flawed genealogical inference [2], [7] is not supported by careful reanalysis of the data [8].

**Figure 1:**
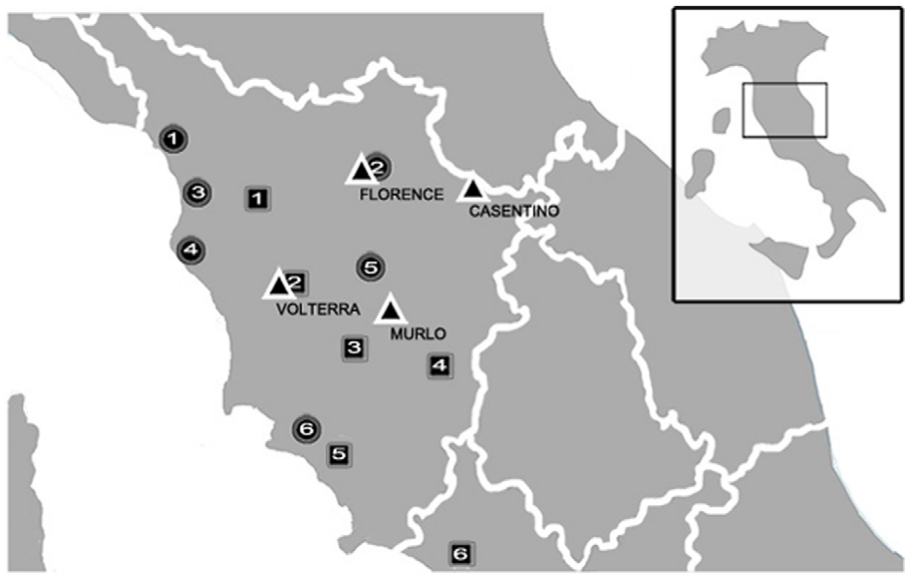
Geographic location of the samples considered in the ABC analysis. Triangles, Contemporary Tuscans (n = 370); Circles, Medieval Tuscans: 1. Massa Carrara (n = 3); 2. Florence, (n = 10); 3. Pisa, (n = 6); 4. Livorno, (n = 3); 5. Siena, (n = 4); 6. Grosseto (n = 1); Squares, Etruscans: 1. Castelfranco di Sotto (n = 1); 2. Volterra (n = 3); 3. Casenovole (n = 10); 4. Castelluccio di Pienza (n = 1); 5. Magliano/Marsiliana (n = 6); 6. Tarquinia (n = 9).

What previous studies overlooked is the potential genetic effect of population subdivision. If most Etruscans’ descendants lived in isolated communities in the last 2,000 years, their DNAs may still persist in some localities, but will escape detection unless they are sought at the appropriate (i.e., smaller) geographical scale. Indeed, previous work in another area of Italy [9] showed that modern populations separated by only tens of kilometers can differ sharply in their genealogical relationships with ancient populations. To investigate in greater geographical detail the biological relationships between contemporary and ancient populations, we thus sampled multiple burials in classical Etruria. MtDNA was extracted from bones, amplified and sequenced by a combination of classical methods and Next Generation Sequencing. After adding these sequences to the other Etruscan sequences produced in our lab [3] we compared them through methods of Approximate Bayesian Computation with those of relevant ancient and modern human populations. These include Medieval Tuscans (n = 27) [5], contemporary Tuscans from three sites in historical Etruria (Casentino, n = 122; Murlo, n = 86; Volterra, n = 114) [2] and from Florence [10] (n = 48) (Figure 1). The sample from Florence here represents a control, since no special relationships is expected between the DNAs of the Etruscans and those of the inhabitants of a large city, after millennia of immigration.

We thus tried to address two questions, namely (1) whether an analysis at the small geographical scale can provide evidence of a genealogical continuity between the Etruscans and some current inhabitants of historical Etruria, and (2) whether the observed degree of genetic resemblance between modern inhabitants of Tuscany and Western Anatolia has anything to do with the Etruscans’ origins. To answer, for each modern population we designed and compared three demographic models differing for the genealogical relationships with the ancient samples (see Material and Methods for details). We identified the model best fitting each set of the observed data, and then we moved to estimating, under an isolation-with-migration (IM) framework, the separation time between Tuscan and Anatolian populations [11], evaluating whether the estimated time can be reconciled with an Etruscan origin in Anatolia and a subsequent migration in Italy around the 8^th^ century BC.

## Results

### Ancient DNA Sequences

We could obtain amplifiable DNA from 14 Etruscan specimens. Four of them, from Tarquinia, were analyzed in 2004 but were still unpublished. Ten samples come from 18 initial bone samples (each represented by two fragments of the right tibia) from a 3^rd^ century BC multiple burial in Casenovole, Southern Tuscany. The bones were freshly excavated and collected according to the most stringent ancient DNA criteria (see Materials and Methods) by one of us (EP); they can safely be regarded as belonging to different individuals. After a first round of DNA extraction, the 18 Casenovole samples were subjected to multiple PCRs, cloning and cycle sequencing. In ten of them we could determine the sequence of the complete mtDNAhypervariable region I (hereafter: HVR-I), whereas the remaining eight gave no results (Figure S1). Their final consensus sequences (Table S1) were determined by comparing results obtained using the standard procedures (575 clones overall) and Next Generation Sequencing (127,837 reads) (Figure S2). We added to these the sequences of four individuals from Tarquinia, (GenBank accession numbers: bankit1285669 GU186064; bankit1285680 GU186065; bankit1285699 GU186066; bankit1285702 GU186067).

### The Etruscans in the Context of Modern and Ancient Genetic Diversity

We analyzed four non-overlapping datasets (Table 1). The ETR dataset comprises the 14 newly produced DNA sequences, along with 16 already available sequences from necropoleis in historic Etruria [3]; individuals from geographically distant Etruscan populations, Adria and Capua, were excluded. The TUS dataset comprises four modern Tuscan populations, i.e. Casentino, Murlo, Volterra and Florence; the last mentioned is a forensic sample, representing random members of a large city, to the exclusion of recent immigrants (Figure 1). In addition, this dataset includes a sample of Medieval Tuscans from Guimaraes et al. [5]. Finally, the ANC dataset and the EUR dataset include, respectively, data on ancient and modern populations from Europe and from the Near East.

**Table 1:** A synopsis of the datasets analyzed.

Dataset	N populations	N individuals	Notes
**ETR**	1	30	Etruscan sequences from the present paper and from Vernesi et al. (2004)
**TUS**	5	397	Medieval and modern sequences from Tuscany
**EUR**	52	4,910	Modern European sequences
**ANC**	9	190	Ancient European sequences

**Figure 2:**
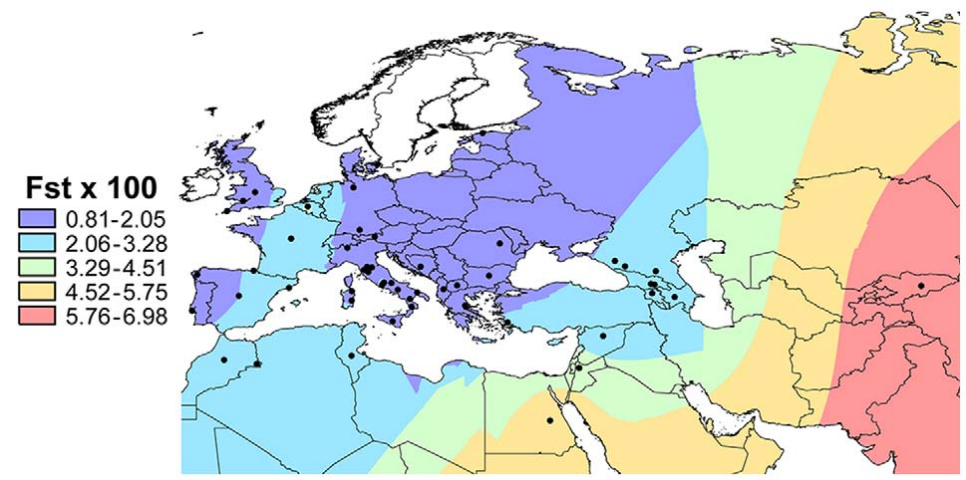
Genetic distances (percent F_ST_ values) between the Etruscan and modern population samples. Different colors represent different levels of genetic differentiation from the Etruscans.

In Table 2 we show several statistics summarizing genetic variation in the ETR and TUS datasets. Estimates of the internal genetic diversity of the Etruscans, as expressed by their mean pairwise difference (2.966±1.560) and by haplotype diversity (0.943±0.032), appear close to those obtained in Vernesi et al. [3] using a partly different dataset. We also calculated two measures of genetic distance between the Etruscans (ETR) and modern populations (EUR), namely Wright’s pairwise Fst and allele sharing, the latter measured as the fraction of modern sequences also observed in the Etruscan sample (Figure S3). A general decline of genetic resemblance with geographic distance is evident (Figure 2).

**Table 2:** Statistics summarizing intra-(A) and inter- (B) population genetic diversity.

A						
	Etruscans	Medieval	Casentino	Murlo	Volterra	Florence
**Number of sequences**	30	27	122	86	114	48
**Number of distinct Haplotypes**	21	14	72	59	57	40
**Mean pairwise difference**	2.966	1.972	4.105	4.278	3.850	4.152
**Haplotype diversity**	0.943	0.860	0.976	0.975	0.955	0.980
**Segregating sites**	24	14	62	64	58	48
**B**						
**Fst**						
	**Etruscans**	**Medieval**	**Casentino**	**Murlo**	**Volterra**	**Florence**
**Etruscans**	0.000	0.015	0.020	0.010	0.012	0.014
**Medieval**	0.015	0.000	0.020	0.015	0.013	0.022
**Allele sharing**						
**Etruscans**	1.000	0.238	0.333	0.143	0.238	0.095
**Medievals**	0.357	1.000	0.500	0.214	0.429	0.143

These values were used in the ABC analysis. Allele sharing was calculated as the number of alleles shared between pairs of populations, over the total number of alleles in the ancient sample.

**Figure 3:**
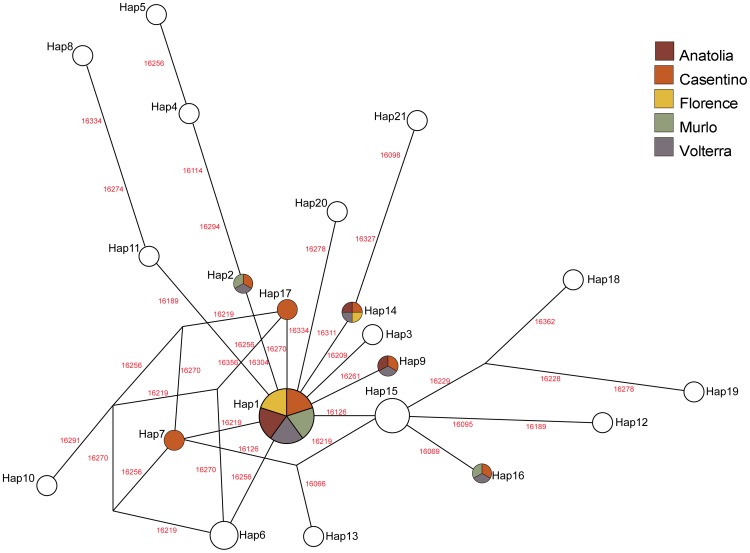
Median-joining network of the Etruscans’ haplotypes. The width of the circles is proportional to the frequency of that haplotype in the Etruscan sample; the labels on the edges of the network indicate the position of the nucleotide substitution in the mtDNA reference sequence. The colour of each haplotype represents whether that sequence is also present in five modern populations from Tuscany and Anatolia.

Among the 30 Etruscan individuals (ETR dataset) we observed 21 different sequences with 24 variable sites (Table 2); the network describing the relationship among the Etruscans’ haplotypes is reported in Figure 3. Comparisons with 52 modern populations in the TUS and EUR datasets (listed in Table S2) show that 11 of these sequences are shared with at least one of 4,910 individuals from Western Eurasia and the Southern Mediterranean shore (Table S1). The Etruscan sample falls within the range of contemporary genetic variation (EUR dataset, Figure S4A, S4B). In the comparison with the samples of the ANC dataset, the Etruscans appear to fall very close to a Neolithic population from Central Europe and to other Tuscan populations; geographically distant Bronze and Iron-age samples, from Iberia and Sardinia, appear genetically differentiated from the Etruscans (Figure S4C).

### Genealogical Relationships between the Etruscans and Contemporary Populations

**Figure 4:**
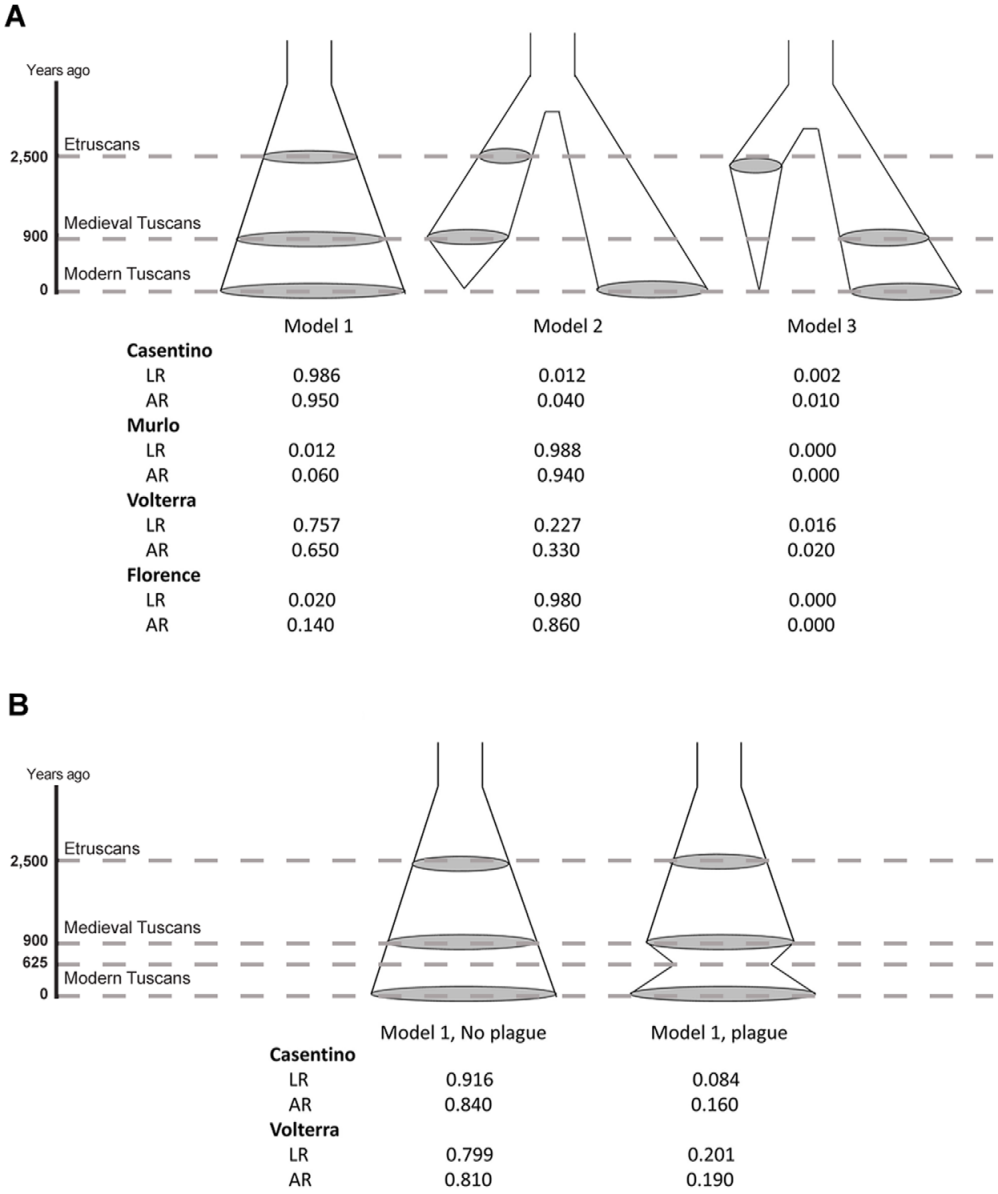
Alternative models of the genealogical relationships among past and present populations, and their posterior probabilities. Shaded areas represent the modern population (at 0 years ago on the Y axis), the Medieval population (900 years ago) and the Etruscans (at 2,500 years ago). Model 1 assumes genealogical continuity between ancient and modern samples, Model 2 assumes continuity only between Etruscan and Medieval individuals, and in Model 3 the Etruscan lineage separates from the lineage leading to Medieval and Modern Tuscans. Under each model is the proportion of the best-fitting simulations supporting it, for the four modern populations considered, using the acceptance rejection (AR) and logistic regression (LR) methods [43]. (A) Comparison among Models 1–3 for four modern Tuscan populations. (B) Comparison of the fit of Model 1, with and without a bottleneck corresponding to the Plague epidemics at 625 BP [12].

We investigated the genealogical relationships between ancient and contemporary samples by Approximate Bayesian Computation (ABC), a set of methods to fit complex evolutionary models to genetic data. We proceeded in 5 steps, namely: (i) we defined 3 alternative models of the genealogical relationships between ancient and current inhabitants of Tuscany (TUS dataset) (Figure 4A); (ii) we generated by serial coalescent simulation millions of gene genealogies for each model; (iii) we summarized genetic diversity in the observed and simulated data by the same set of statistics (Table 2); (iv) by comparing these statistics in the observed and simulated data, we selected a set of simulations best reproducing variation in the data (the number of simulations retained depends on the criterion chosen for the model selection: 100 for the simple rejection procedure and 50,000 for the weighted multinomial logistic regression); and (v) we estimated the models’ posterior probabilities (*PP*) by counting how many of the selected simulations were generated under each model (normalizing so that the sum of *PP*s for all models is equal to 1). Demographic (population sizes) and evolutionary (mutation rates) parameters were explored in the simulations within a broad range of possible values defined by priors, and finally estimated from the simulated data.

In total, 24 million simulations were run (1 million for each of 3 models, 4 modern populations in the TUS dataset, and 2 demographic scenarios, respectively including or not including a bottleneck at the time of the Medieval plague epidemics [12]).

We found evidence for genealogical continuity all the way from Etruscan to current times in two contemporary populations (Figure 4A); the posterior probability (*PP*) of Model 1 was between 0.65 and 0.76 for Volterra and 0.95 and 0.99 for Casentino, and this result did not change considering different numbers of best-fitting simulations (say, 500 instead of 100, or 100,000 instead of 50,000). Similar results were obtained incorporating in the model a recent population bottleneck (Figure S5), although an explicit comparison between models with and without plague favoured the latter (Figure 4B). At any rate, the relative success of the models does not depend on the presence of a bottleneck in the late Middle Age. Therefore, this event was not considered in subsequent analyses.

By contrast, for Murlo and Florence, Model 2, with the modern DNAs occupying a distinct branch of the genealogical tree with respect to Etruscans and Medieval Tuscans, was shown to be 7 to 99 times more likely than any alternative model (*PP* between 0.86 and 0.99) (Figure 4A); Model 3 received essentially no support. Choosing different sets of statistics to summarize the data did not change the essence of the results.

We then asked whether there is enough power in the data for these models to be discriminated. To answer, we generated by simulation (separately for Casentino, Murlo, Volterra and Florence) 1,000 pseudo-observed datasets according to each model analyzed (Models 1–3), with parameter values randomly chosen from the correspondent prior distribution. We analyzed these pseudo-observed data with the standard ABC procedure, and counted the fraction of cases in which the model used to generate the data was not recognized, or Type I error. We found that Type I error was always ≤0.08 and that the model emerging from the analysis of the observed data (Model 1 for Casentino and Volterra, Model 2 for Murlo and Florence) was correctly identified in at least 95% of cases (Table 3).

**Table 3:** Type I errors for the 3 Models in the 4 Tuscan samples.

Simulated Model					
**CASENTINO**					
		**MOD 1**	**MOD 2**	**MOD 3**	**Type I error**
	**MOD 1**	**0.98**	0.00	0.02	0.02
	**MOD 2**	0.01	**0.99**	0.00	0.01
	**MOD 3**	0.02	0.00	**0.98**	0.02
**MURLO**					
		**MOD 1**	**MOD 2**	**MOD 3**	**Type I error**
	**MOD 1**	**0.95**	0.01	0.04	0.05
	**MOD 2**	0.02	**0.98**	0.00	0.02
	**MOD 3**	0.07	0.00	**0.93**	0.07
**VOLTERRA**					
		**MOD 1**	**MOD 2**	**MOD 3**	**Type I error**
	**MOD 1**	**1.00**	0.00	0.00	0.00
	**MOD 2**	0.07	**0.93**	0.00	0.07
	**MOD 3**	0.05	0.00	**0.95**	0.05
**FLORENCE**					
		**MOD 1**	**MOD 2**	**MOD 3**	**Type I error**
	**MOD 1**	**0.92**	0.03	0.05	0.08
	**MOD 2**	0.04	**0.95**	0.01	0.05
	**MOD 3**	0.05	0.01	**0.94**	0.06

For each of the modern populations listed on the Y axis, data were simulated according to three models and attributed by the LR procedure to one of the models on the X-axis. The power of the procedure in recovering the correct model is represented by the rates of correct attribution (along the main diagonal; shaded cells); the last column (Type I error) represents the fraction of cases in which the correct model was not identified.

Under Model 1, archaic population sizes appear small in both Tuscan populations, with an exponential growth starting around 10,000 years ago for Casentino and 16,500 years ago for Volterra (Figure S6). The estimated mutation rate (around 0.3 mutational events per million years per nucleotide) is in agreement with previous independent reports [9], [13]. In general, all the parameters appear well estimated; indeed, their R^2^value are always higher than 0.1, an empirical figure generally accepted to be the value beyond which an estimate may be considered reliable [14]. We note that the posterior distribution of the modern effective population sizes drives to the upper limit of the priors (Figure S6). This has also been observed in previous comparable studies [15]–[17] and reflects the fact that the estimated population size is basically a function of the existing genetic diversity. Clearly, immigration processes have introduced new haplotypes in populations that we had to model as genetically isolated; the resulting excess of diversity is reflected in an increase of the estimated population size. However, in simulations based on the parameters estimated for Model 1 (posterior predictive tests) we succeeded in generating patterns of variation fully compatible with the observed variation; the model’s *P*-values (0.332 for Casentino, 0.380 for Volterra) show that the statistics estimated from the observed and simulated data do not differ significantly, and imply that problems related with the estimation of modern population sizes did not undermine the general validity of our approach.

### An Etruscan Origin in Anatolia?

**Figure 5:**
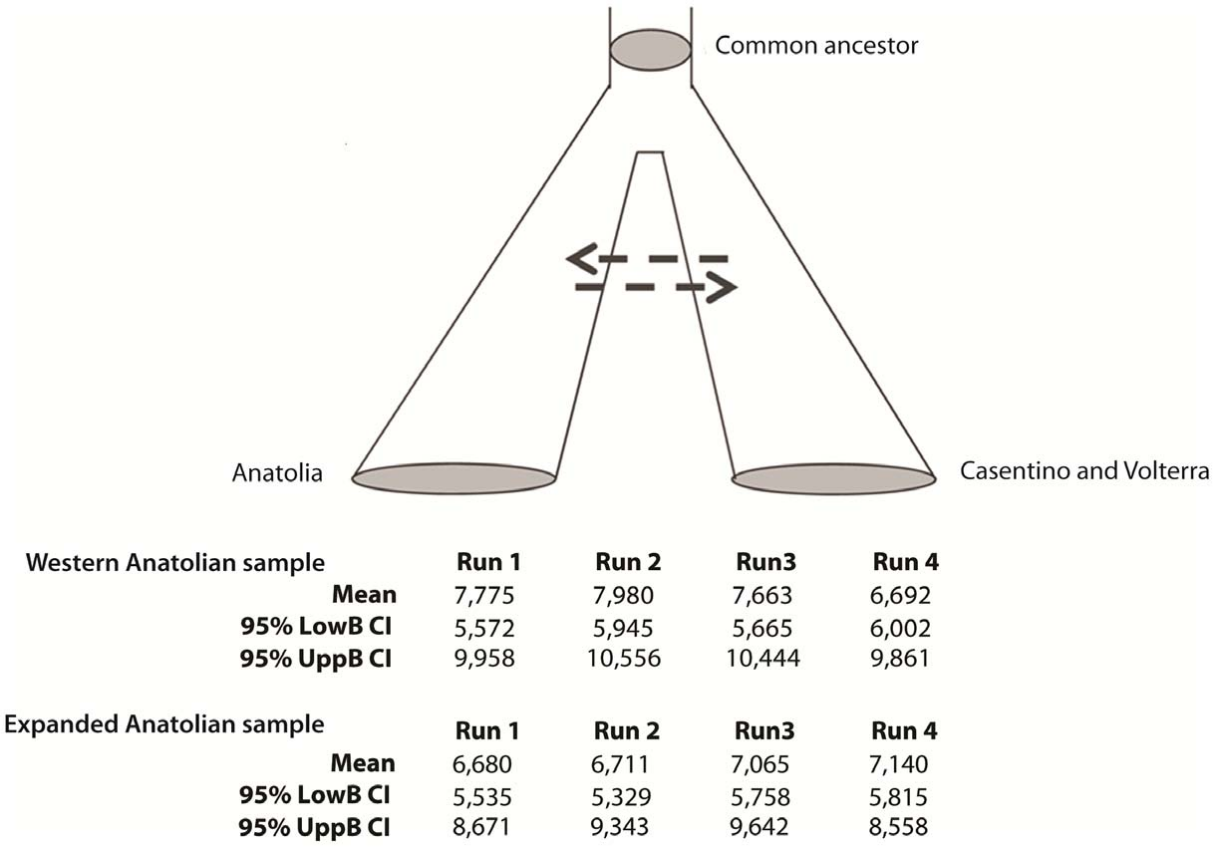
Separation time estimated by the IM model. Estimation of the separation time between the gene pools of Anatolians (whether only Western Anatolians, or the expanded sample) and contemporary Tuscans (Casentino and Volterra). Means, upper bound and lower bound of the 95% credible intervals in four independent runs, obtained fixing the migration rate (indicated by dashed arrows) at 0, with mutation rate = 0.003 and assuming that the proportion of the ancestral population is equal in each descendant population (i.e. s = 0.5). Each analysis consisted of five coupled Markov chains, and 10,000,000 steps. Any degree of gene flow between the ancestors of Anatolians and Tuscans results in an increase of the estimate of the time since the population separation.

Going back to the issue of the Etruscans’ origins, if the genetic resemblance between Turks and Tuscans reflects a common origin just before the onset of the Etruscan culture, as hypothesized by Herodotus and as considered in some recent studies [2], [6], [18], we would expect that the two populations separated about 3,000 years ago. To discriminate between the potentially similar effects of remote common origin and recent gene flow, we ran four independent analyses based on the IM method [19], [20]. In the model we tested, the two populations originate from a common ancestor, and may or may not exchange migrants after the split (Figure S7A). Assuming an average generation time of 25 years [16], [21] and no migration after the split from the common ancestors, the most likely separation time between Tuscany and Western Anatolia falls around 7,600 years ago, with a 95% credible interval between 5,000 and 10,000 (Figure 5). These results are robust to changes in the proportion of members of the initial population being ancestral to the two modern populations (Figure S7B). We also considered an expanded Anatolian sample (total sample size = 123 [11], [22]) coming from all over Turkey, to test whether a founder effect might have enhanced the role of the genetic drift in the previous analysis, inflating the divergence time estimates; the resulting distributions of separation times completely overlapped with those previously estimated, with a lower bound of the 95% credible interval never smaller than 5,300 years ago (Figure 5).

For these tests we chose the mutation rate (μ) estimated from the data in the previous ABC analyses (very close to the figure accounting for the time-dependency of the mitochondrial molecular clock [13], μ = 0.003). Tests were also run using the value incorporating a correction for the effects of purifying selection [23] (μ = 0.0014), always finding that it results in a further increase of the estimated separation times (Figure S7B). Only assuming very high mutation rates, at least twice as large as estimated in Henn et al. [13], was it possible to obtain separation times <5,000 years (Figure S7B). With both Anatolian samples, any degree of gene flow after separation between the ancestors of Tuscans and Anatolians resulted in more remote separation times.

## Discussion

MtDNA data give much stronger support to a model of genetic continuity between the Etruscans and some Tuscans than to any other model tested, characterized by plausible population sizes and mutation rates. However, this clear picture emerges only when modern Tuscan communities are separately considered, highlighting the importance of population structure even at the small geographical scale. In a previous analysis of smaller samples we found no evidence of genealogical continuity since Etruscan times [5]. In this study, the larger sample sizes allowed us to separately investigate the relationships of each modern population with the Etruscans. A model of genealogical continuity across 2,500 years thus proved to best fit the observed data for Volterra, and especially Casentino, but not for another community dwelling in an area also rich with Etruscan archaeological remains (Murlo), nor (as expected) for the bulk of the current Tuscan population, here represented by a forensic sample of the inhabitants of Florence. Therefore, the present analysis indicates that the Etruscan genetic heritage is still present, but only in some isolates, whereas current Tuscans are not generally descended from Etruscan ancestors along the female lines. It also shows that there is no necessary correlation between the presence of archaeological remains and the biological roots of the inhabitants of the areas where these remains occur. Because Medieval Tuscans appears directly descended from Etruscan ancestors, one can reasonably speculate that the genetic build-up of the Murlo and Florence populations was modified by immigration in the last five centuries.

As for the second question, the IM analysis shows that indeed there might have been a genealogical link between modern Tuscans and the inhabitants of what Herodotus considered the Etruscans’ homeland, Western Anatolia. However, even under the unrealistic assumption of complete reciprocal isolation for millennia, the likely separation of the Tuscan and Anatolian gene pools must be placed long before the onset of the Etruscan culture, at least in Neolithic times; if isolation was incomplete, the estimated separation must be placed further back in time. Consistent with this view is the observation that Etruscan and Neolithic mtDNAs are close to each other in the two-dimensional plot of Figure S4C; however, a formal test would be necessary to draw firm conclusions from the simple observation of a genetic similarity. Separation times were very close when estimated both using a sample from Western Anatolia, and an expanded sample including individuals from much of Anatolia, and so the choice of the Anatolian population does not seem to affect the results of this analysis.

A general problem in ancient human DNA studies is the quality of the data; errors resulting from contamination, or from poor preservation of DNA in the specimens, are common. However, there are several reasons to be confident that the Etruscan sequences obtained in this study are authentic: (i) bones were recovered from burials according to the most stringent existing procedures and sent directly to the ancient DNA laboratory without manipulations; (ii) the mtDNA HVR-I motifs of the people who came in contact with the bones at any stage of the analysis do not match those obtained from the ancient samples (Table S1); (iii) the ancient samples were typed following the most stringent standard criteria for ancient DNA authentication; (iv) we used two different sequence determination procedures (classical methodology and high throughput methodology) and the results obtained from different extractions and different sequencing methodologies are concordant except in the regions of homopolymeric strings ≥5 bp that are problematic for the 454 pyrosequencing technology; in these cases, consensus sequences were determined considering only the results of the standard sequencing procedure; (v) sequences make phylogenetic sense, i.e. do not appear to be combinations of different sequences, possibly suggesting contamination by exogenous DNA.

Using such ancient DNA data for testing complex evolutionary models has become possible with the development of ABC and other recent Bayesian inference methods [24], [25]. These models, albeit more articulate than those that can be tested otherwise, are still a necessarily schematic representation of the processes affecting populations in the course of millennia. Many phenomena that could not be incorporated in the models, such as immigration from other sources or additional demographic fluctuations, most likely occurred and left a mark in the patterns of genetic diversity. In addition, specific phenomena may have involved mostly or exclusively males, resulting in genetic changes that are not recorded in mtDNA variation. Still, if we rule out the unlikely hypothesis that the Etruscans’ and their descendants’ population history was radically different for males and females, the picture emerging from this study is rather clear. The additional tests we ran (Type I error, Table 3) show that, at these sample sizes, we had a high probability to identify the correct evolutionary model.

As also suggested by the analysis of skull diversity [26], contacts between people from the Eastern Mediterranean shores and Central Italy likely date back to a remote stage of prehistory, possibly to the spread of farmers from the Near East during the Neolithic period [27], [28], but not necessarily so (we only estimated a minimum separation time between gene pools). At any rate, these contacts occurred much earlier than, and hence appear unrelated with, the onset of the Etruscan culture (Figure 5). We conclude that no available genetic evidence suggests an Etruscan origin outside Italy. While their culture disappeared from the records, the Etruscans’ mtDNAs did not; traces of this heritage are still recognizable. However, most current inhabitants of the ancient Etruscan homeland appear descended from different ancestors along the female lines, as clearly shown by the analysis of the urban (Florence) sample. Genetic continuity since the Etruscan’s time is still evident only in relatively isolated localities, such as Casentino and Volterra.

## Materials and Methods

### DNA Extraction and Characterization of the Etruscan Samples

We obtained 18 bone samples (each represented by two fragments of the right tibia) from a multiple burial from Casenovole, Southern Tuscany, near Grosseto. Their approximate age, based on archaeological evidence, is the 3^rd^ century BC. The permit to genetically characterize these fossil samples came from Soprintendenza Archeologica per la Toscana (Archaeological Authority for Tuscany), Siena. The bone fragments were freshly excavated and collected according to the most stringent ancient DNA criteria [29] by one of us (EP) and can safely be regarded as belonging to different individuals (Minimum number of individuals estimated in the burial = 21). These fragments were processed in the ancient DNA facilities at the University of Florence using standard ancient DNA procedures [30]. After a first round of DNA extraction, the samples were subjected to multiple PCRs, cloning and cycle sequencing.

In a successive step, DNA was independently reextracted from the samples that had given positive results in the previous analysis. In this case, after multiple PCRs, the amplicons were not cloned but ligated to the appropriate adaptor sequences and directly sequenced with 454/Roche technology. Low Molecular Weight DNA (LMW DNA) 454/Roche protocol was applied and a final procedure modification was added to increase the recovery of a single stranded library [31]. Libraries were quantitated using a quantification Real Time PCR (qPCR) by KAPA Library Quant Kits (KAPA Biosystems, MA, USA). Samples libraries were independently amplified on beads by emulsion PCR (emPCR), then enriched and counted beads were loaded onto 454/Roche PicoTiterPlate (PTP) divided in 16 regions. Sequencing was performed as in 454/Roche protocol and the obtained reads were filtered and mapped using the Cambridge reference sequence [32]. For each sample and amplicon, a masking procedure allowed to remove primer sequences from the reads and obtain a multi-alignment using the 454/Roche Amplicon Variant Analysis (AVA) software. A consensus was generated by custom scripting and then mapped on the mitochondrial DNA reference sequence (GenBank accession number: J01415). Complete mtDNA HVR-I sequences could be retrieved in all samples. At each site the most frequent nucleotide was observed in a range of 97.7–98.8% of the reads in the different samples. Unmapped reads were then analyzed in order to characterize them and we found that they are mostly primer dimers. Final consensus sequences of the 10 samples were determined by comparing results obtained from both standard procedures (575 Clones) and Next Generation Sequencing (127,837 reads).

Four additional samples from Tarquinia, sequenced in 2004, but never published so far, brought to 14 the total of Etruscan samples typed for this study.

### Ancient and Modern mtDNA Diversity

In all statistic analyses, we replaced the nucleotides occupying position 16180–16188 and 16190–16193 with the nucleotides in the CRS, because they contain two stretches of Adenines and Citosines known to result in apparent length polymorphism of the mtDNA sequence [33], [34]. Summary statistics were estimated by Arlequin ver. 3.5.1 [35]. The Fst values between the populations in the EUR dataset and the Etruscans were interpolated in a map representing using the Spatial Analyst extension in ArcGIS 10 (ESRI; Redlands, CA, USA) using the Kriging procedure. Genetic distances between the Etruscans and each population in the ANC, TUS and EUR datasets were visualized by Multidimensional Scaling (MDS), using the *cmdscale* function in the R environment [36].

### Approximate Bayesian Computation

Inferring demographic and evolutionary processes from genetic data requires the testing of models which are often too complex for their likelihoods to be derived. Approximate Bayesian Computation (ABC) [37] offers a valid alternative. Summary statistics estimated from the data are compared with those generated by simulation, and posterior distributions of the models’ parameters can be approximated by simulating large numbers of gene genealogies. We generated gene genealogies in which individuals are sampled at different moments in time using the Bayesian version of SERIALSIMCOAL [38]. At every iteration, the parameters of the model (population sizes, mutation rates, timing of demographic processes) were considered as random variables, and their values were extracted from broad prior distributions; ages and sizes of the samples were equal to those of the observed samples. We then calculated a Euclidean distance between observed and simulated statistics, and we ordered the simulations according to this distance. In total, 24 million simulations were run (1 million for each of 3 models, 4 modern populations in the TUS dataset and two demographic scenarios, respectively including or not including a recent bottleneck). All the procedures were developed in the R environment [36] using scripts from [39]. We selected the summary statistics via PCA, keeping for the ABC analysis those statistics which have shown to be more correlated with the parameters’ variance (Table S2).

### Demographic Models and Priors

The three demographic models tested differ for the relationships between modern and ancient samples (Figure 4); under each model, each population in the TUS dataset was independently compared with the Etruscan and Medieval populations. All prior distributions were uniform and wide. The effective modern population size ranged between 100 and 200,000; for the time of the onset of the expansion (under Model 1) and the separation time (under Models 2 and 3) the priors ranged from 101 (one generation before the Etruscans) to 1,500 generations ago. Priors for the mutation rate encompassed the low value estimated from phylogenies [40], and the high value estimated from pedigrees [41], from 0.0003 to 0.0075 mutations per generation for HVR-I. The Medieval and the Etruscan effective population sizes were extracted from a prior distribution spanning from 100 to 50,000, as suggested in Guimaraes et al. [5]. Ancestral population sizes varied from 5 to 6,000 individuals. The entire procedure was repeated under a demographic scenario including a population bottleneck corresponding to the 14^th^ century plague epidemics, in which an estimated one-third of the population was lost [42].

### Model Selection and Parameter Estimation

The posterior probabilities of the 24 combinations of models (3), modern populations (4) and demographic scenarios (2), were calculated either: (i) by a simple rejection procedure (AR) [43] for which we retained the 100 simulations associated with the shortest distance between observed and simulated statistics [44]; or (ii) by a weighted multinomial logistic regression (LR) [44] for which we retained the 50,000 simulations generating the shortest distance between the observed and simulated statistics. In both cases, we normalized the PPs so that their sum for all models being compared is 1. The parameters of the best-fitting model were estimated from the 2,000 simulations closest to the observed dataset, after a *logtan* transformation of the parameters [45] and according to Beaumont [37].

### Additional Tests: Type I Error and Posterior Predictive Tests

We estimated the probability that the true null hypothesis be rejected by evaluating the Type I Error, i.e. the proportion of cases in which 1,000 pseudo-datasets generated under each model are not correctly identified by the ABC analysis. In addition, to test whether the data can be actually reproduced under a specific demographic model, we carried out a posterior predictive test [9], [25]. For that purpose, we simulated 10,000 datasets according to the model with the highest probability using the estimated posterior parameter distribution, and we calculated a posterior predictive P-value for each statistic; these probabilities were then combined into a global P-value, taking into account their non-independence [46].

### The Isolation with Migration (IM) Model

We estimated the likely separation time between the Tuscan and Anatolian gene pools by Isolation with Migration (IM), a method generating posterior probabilities for complex models in which populations need not be at equilibrium [19]. Seven parameters were estimated from the data, namely the size of the ancestral and daughter populations (*N_A_, N_1_, N_2_*), the rates of gene flow between daughter populations (*m_1_, m_2_*), the time since the split (*t*), and the proportion of the members of the ancestral population giving rise to the first daughter population (*s*) [47]. Because any degree of genetic exchange increases the *t* estimate, after some preliminary tests we set to 0 the values of *m_1_* and *m_2_*. Most tests were run fixing the mutation rate at the value estimated in the ABC analysis (0.003 mutational events per locus per generation), but we repeated the whole IM analysis with both lower and higher values (respectively, 0.0014 and 0.0060 mutational events per locus per generation; [13], [23]) under a Hasegawa-Kishino-Yano (HKY; [48]) mutational model with inheritance scalar 0.25, as recommended for mtDNA data. For each mutation rate tested we ran several analyses starting from different random seeds, in order to assess the consistency of the results; moreover, to improve the exploration of the parameters’ space, and thereby the convergence, we coupled the Markov chains, running simultaneously 5 chains per run.

## Supporting Information

Figure S1 **Amplicons of the 10 sequences from Casenovole.** DNA sequences from the575 clones analysed for the 10 Casenovole Etruscan samples. The sequences of the external primers are not reported in the figure. The Cambridge reference sequence with the numbering of the nucleotide positions is at the top. Nucleotides identical to the Cambridge reference sequence are indicated by dots. The clones are identified by a code (from S1 to S17, indicating the individual), the first number is the extraction, the second number is the PCR.
(PDF)

Figure S2 **Results of the mapping step for the 10 Etruscan samples analyzed.** (A) The number of sequences that map to the reference and those that do not map is plotted as a histogram. Some samples had a large amount of unmapped reads that were afterwards characterized as primers’ dimers. (B) Frequency distribution (% on the Y axis) of the frequency of the most frequent nucleotide for the 10 Etruscan samples analyzed (the upper limits of the % intervals are reported in the legend). For example, in sample S1 at around 84% of the positions the frequency of the most frequent allele among reads is between 99% and 100%.
(PDF)

Figure S3 **Measures of genetic distance.** Allele sharing (A) and Fst (×100) (B) in 52 modern populations of Western Eurasia and the Mediterranean basin. Population labels and sample sizes are provided in Table S2. Allele sharing estimated as the number of sequences shared between Etruscans and every modern population, divided by the sample size of the modern sample.
(TIF)

Figure S4 **Multi Dimensional Scaling.** Multi Dimensional Scaling summarizing genetic affinities between the Etruscans and (A) 52 modern populations of Western Eurasia and the Mediterranean basin; (B) Medieval and modern Italian populations; (C) 9 ancient populations of Europe. Population labels and sample sizes are provided in Table S2.
(PDF)

Figure S5 **Results of model selection.** Results of model selection with or without a bottleneck representing the plague epidemics at 625 BP, in Casentino, Murlo and Volterra. Dashed lines represent the presence of plague epidemic that killed one third of the population. For each sample we report the posterior probabilities calculated comparing Models 1–3, either considering or disregarding this demographic event.
(PDF)

Figure S6 **Parameter estimates and posterior distributions under Model 1, for Casentino (A) and Volterra (B).** Upper panels: Prior distributions (all the priors were uniform), median and mode estimates, the 95% of the highest posterior density (lower and upper bound), and coefficient of determination R^2^. The time is expressed in years, the mutation rate in number of mutational events per generation per locus. Lower panels: histograms and smoothed distributions of the parameters estimated.
(PDF)

Figure S7 **IM model (A) and estimates (B) for the separation time between Anatolians and Tuscans.** N_1_ and N_2_: modern population size; N_A_: ancestral population size; m_1_ and m_2_: migration rates; s: proportion of the ancestral population that founds descendent population 1; t: separation time. Different mutation rates and proportions of the ancestral population founding the descendant populations were considered.
(PDF)

Table S1 **Consensus HVR-I Etruscans mtDNA and sequences of all the investigators.** Upper panel: Consensus HVR-I mtDNA sequences in 30 individuals from historical Etruria. Tarq represents individuals from Tarquinia, Cas from Casenovole, Vol from Volterra, Pie from Castelluccio di Pienza, Sot from Castelfranco di Sotto and MM from Magliano and Marsiliana. CRS is the Cambridge reference sequence [32]. The HVR-I motif is the position (−16,000) where substitution were observed, with respect to the CRS; the observed transversions are indicated with a capital letter. The haplotypes shared with EUR dataset are in bold type. For the Casenovole sample, the labels of the individuals used in Figure S1 are between parentheses. Lower panel: Sequences of all the investigators who had direct contact with the ancient specimens.
(DOCX)

Table S2 **Detailed description of the samples in the EUR and ANC datasets.**
(DOC)
