# Supplementary material for: Origins and Evolution of the Etruscans’ mtDNA
Source: PLoS One. 2013 Feb 6;8(2):e55519. doi: 10.1371/journal.pone.0055519 (PMC3566088; doi:10.1371/journal.pone.0055519)
Supplement: Figure S1 — Amplicons of the 10 sequences from Casenovole. DNA sequences from the575 clones analysed for the 10 Casenovole Etruscan samples. The sequences of the external primers are not reported in the figure. The Cambridge reference sequence with the numbering of the nucleotide positions is at the top. Nucleotides identical to the Cambridge reference sequence are indicated by dots. The clones are identified by a code (from S1 to S17, indicating the individual), the first number is the extraction, the second number is the PCR. (PDF) [file pone.0055519.s001.pdf]

```
        11111111111111111111111111111111111111111111111111111111111111111111111111111111111111111111111111111111111111111111111111111111111111111111111111111111111111111111111111
        66666666666666666666666666666666666666666666666666666666666666666666666666666666666666666666666666666666666666666666666666666666666666666666666666666666666666666666666666
        00000000000000000000000000000000000000000000000000000000000000000000000000001111111111111111111111111111111111111111111111111111111111111111111111111111111111111111111111
        22222233333333334444444444555555555566666666667777777777888888888899999999990000000000111111111122222222223333333333444444444455555555556666666666777777777788888888889999999999
        45678901234567890123456789012345678901234567890123456789012345678901234567890123456789012345678901234567890123456789012345678901234567890123456789012345678901234567890123456789012345678901234567

CRS     TTCTTTCATGGGGAAGCAGATTTGGGTACCACCCAAGTATTGACTCACCCATCAACAACCGCTATGTATTTCGTACATTACTGCCAGCCACCATGAATATTGTACGGTACCATAAATACTTGACCACCTGTAGTACATAAAAACCCAATCCACATCAAAACCCCCTCCCCATGC

S1 1.1  ....................................A.......................................................................ACCATAAATACTTGACCACCTGTAGTAC
S1 1.1  ....................................A.......................................................................
S1 1.1  ............................................................................................................
S1 1.2  ................................................................C...............C...........................
S1 2.1  ............................................................................................................
S1 2.1  ............................................................................................................
S1 2.2  ............................................................................................................
S1 2.2  ............................................................................................................

S1 1.1                                                             CGCTATGTATTTCGTACATTACTGC....................................................................................T.....
S1 1.1                                                                                      ....................................................................................T.....
S1 1.2                                                                                      ....................................................................................T.....
S1 1.2                                                                                      ....................................................................................T.....
S1 2.1                                                                                      .........C..........................................................................T.....
S1 2.1                                                                                      ....................................................................................T.....
S1 2.1                                                                                      ....................................................................................T.....
S1 2.2                                                                                      ....................................................................................T.....
S1 2.2                                                                                      .................................................................................C..T.....

S1 1.1
S1 1.1
S1 1.2
S1 1.2
S1 2.1
S1 2.2
S1 2.2
S1 2.2

S1      ........................................................................................................................................................................T.....

        111111111111111111111111111111111111111111111111111111111111111111111111111111111111111111111111111111111111111111111111111111111111111111111111111111111111111111111
        666666666666666666666666666666666666666666666666666666666666666666666666666666666666666666666666666666666666666666666666666666666666666666666666666666666666666666666
        000000000000000000000000000000000000000000000000000000000000000000000000000011111111111111111111111111111111111111111111111111111111111111111111111111111111111111111
        222222333333333344444444445555555555666666666677777777778888888888999999999900000000001111111111222222222233333333334444444444555555555566666666667777777777888888888899999
        456789012345678901234567890123456789012345678901234567890123456789012345678901234567890123456789012345678901234567890123456789012345678901234567890123456789012345678901234

CRS     TTCTTTCATGGGGAAGCAGATTTGGGTACCACCCAAGTATTGACTCACCCATCAACAACCGCTATGTATTTCGTACATTACTGCCAGCCACCATGAATATTGTACGGTACCATAAATACTTGACCACCTGTAGTACATAAAAACCCAATCCACATCAAAACCCCCTCCCCA

S3 1.1  ............................................................................................................ACCATAAATACTTGACCACCTGTAGTAC
S3 1.1  ............................................................................................................
S3 1.1  ............................................................................................................
S3 1.1  ....................................................................:...........................G...........
S3 2.1  ............................................................................................................
S3 2.1  ............................................................................................................
S3 2.1  ............................................................................................................
S3 2.1  ................................................................................................G...........

S3 1.1                                                             CGCTATGTATTTCGTACATTACTGC....................................................................................T..
S3 1.1                                                                                      ....................................................................................T..
S3 1.1                                                                                      ....................................................................................T..
S3 1.1                                                                                      ....................................................................................T..
S3 1.2                                                                                      ....................................................................................T..
S3 2.1                                                                                      ....................................................................................T..
S3 2.1                                                                                      ....................................................................................T..
S3 2.2                                                                                      ....................................................................................T..
S3 2.2                                                                                      ....................................................................................T..

S3 1.1
S3 1.1
S3 1.1
S3 2.1
S3 2.1
S3 2.2
S3 2.2
S3 2.2

S3      ........................................................................................................................................................................T..
```

```
1111111111111111111111111111111111111111111111111111111111111111111111111111111111111111111111111111111111111111111111111111111111111111111111111111111111111111111111111111111111111111111
6666666666666666666666666666666666666666666666666666666666666666666666666666666666666666666666666666666666666666666666666666666666666666666666666666666666666666666666666666666666666666666
1122222222222222222222222222222222222222222222222222222222222222222222222222222222222222222222222222223333333333333333333333333333333333333333333333333333333333333333333333333333333333333
9900000000001111111111222222222233333333334444444444555555555566666666667777777777888888888899999999990000000000111111111122222222223333333333444444444455555555556666666666777777777788888
8901234567890123456789012345678901234567890123456789012345678901234567890123456789012345678901234567890123456789012345678901234567890123456789012345678901234567890123456789012345678901234

TTACAAGCAAGTACAGCAATCAACCCTCAACTATCACACATCAACTGCAACTCCAAAGCCACCCCTCACCCACTAGGATACCAACAAACCTACCCACCCTTAACAGTACATAGTACATAAAGCCATTTACCGTACATAGCACATTACAGTCAAATCCCTTCTCGTCCCCATGGATGACCCCCCTCAG

.................................C.............................CCTCACCCACTAGGATACCA
...............................................................
...............................................................
...............................................................
...............................................................
...............................................................
...............................................................
...............................................................
...............................................................

                            CAACTATCACACATCAACTGCAA.........................................................................................................................................
                                                   .........................................................................................................................................
                                                   .........................................................................................................................................
                                                   ................................................T........................................................................................
                                                   .........................................................................................................................................
                                                   .........................................................................................................................................
                                                   .........................................................................................................................................
                                                   .........................................................................................................................................

...........................................................................................................................................................................................

1111111111111111111111111111111111111111111111111111111111111111111111111111111111111111111111111111111111111111111111111111111111111111111111111111111111111111111111111111111111111111111111
6666666666666666666666666666666666666666666666666666666666666666666666666666666666666666666666666666666666666666666666666666666666666666666666666666666666666666666666666666666666666666666666
1111122222222222222222222222222222222222222222222222222222222222222222222222222222222222222222222222222223333333333333333333333333333333333333333333333333333333333333333333333333333333333333
9999900000000001111111111222222222233333333334444444444555555555566666666667777777777888888888899999999990000000000111111111122222222223333333333444444444455555555556666666666777777777788888
5678901234567890123456789012345678901234567890123456789012345678901234567890123456789012345678901234567890123456789012345678901234567890123456789012345678901234567890123456789012345678901234

TGCTTACAAGCAAGTACAGCAATCAACCCTCAACTATCACACATCAACTGCAACTCCAAAGCCACCCCTCACCCACTAGGATACCAACAAACCTACCCACCCTTAACAGTACATAGTACATAAAGCCATTTACCGTACATAGCACATTACAGTCAAATCCCTTCTCGTCCCCATGGATGACCCCCCTCAG

.............................................................T.....CCTCACCCACTAGGATACCA
.............................................................T.....
.............................................................T.....
.............................................................T.....
.............................................................T.....
.............................................................T.....
.............................................................T.....
.............................................................T.....
.............................................................T.....

                              CAACTATCACACATCAACTGCAA........T................................................................................................................................
                                                     ........T................................................................................................................................
                                                     ........T................................................................................................................................
                                                     ........T.................................................................C..............................................................
                                                     ........T................................................................................................................................
                                                     ........T................................................................................................................................
                                                     ........T................................................................................................................................
                                                     ........T................................................................................................................................

.............................................................T................................................................................................................................
```

```
        111111111111111111111111111111111111111111111111111111111111111111111111111111111111111111111111111111111111111111111111111111111111111111111111111111111111111111111111111
        666666666666666666666666666666666666666666666666666666666666666666666666666666666666666666666666666666666666666666666666666666666666666666666666666666666666666666666666666
        000000000000000000000000000000000000000000000000000000000000000000000000000011111111111111111111111111111111111111111111111111111111111111111111111111111111111111111111111
        222222333333333344444444445555555555666666666677777777778888888888999999999900000000001111111111222222222233333333334444444444555555555566666666667777777777888888888899999
        456789012345678901234567890123456789012345678901234567890123456789012345678901234567890123456789012345678901234567890123456789012345678901234567890123456789012345678901234

CRS     TTCTTTCATGGGGAAGCAGATTTGGGTACCACCCAAGTATTGACTCACCCATCAACAACCGCTATGTATTTCGTACATTACTGCCAGCCACCATGAATATTGTACGGTACCATAAATACTTGACCACCTGTAGTACATAAAAACCCAATCCACATCAAAACCCCCTCCCCA

S4 1.1  ..........................................................................................A.................ACCATAAATACTTGACCACCTGTAGTAC
S4 1.1  ..........................................................................................A.................
S4 1.2  ..........................................................................................A.................
S4 1.2  ..........................................................................................A.................
S4 1.2  ..........................................................................................A.................
S4 2.1  ..........................................................................................A.................
S4 2.1  ..........................................................................................A.................
S4 2.1  ..........................................................................................A.................

S4 1.1                                                             CGCTATGTATTTCGTACATTACTGC......A.............................................................................T..
S4 1.1                                                                                       .....A.............................................................................T..
S4 1.1                                                                                       .....A.................................................GG..........................T..
S4 1.2                                                                                       .....A.............................................................................T..
S4 1.2                                                                                       .....A.............................................................................T..
S4 1.2                                                                                       .....A.............................................................................T..
S4 2.1                                                                                       .....A.............................................................................T..
S4 2.1                                                                                       .....A.............................................................................T..
S4 2.1                                                                                       .....A.............................................................................T..

S4 1.1
S4 1.1
S4 1.2
S4 1.2
S4 2.1
S4 2.1
S4 2.2
S4 2.2

S4      ..........................................................................................A.............................................................................T..

        111111111111111111111111111111111111111111111111111111111111111111111111111111111111111111111111111111111111111111111111111111111111111111111111111111111111111111111111111
        666666666666666666666666666666666666666666666666666666666666666666666666666666666666666666666666666666666666666666666666666666666666666666666666666666666666666666666666666
        000000000000000000000000000000000000000000000000000000000000000000000000000011111111111111111111111111111111111111111111111111111111111111111111111111111111111111111111111
        222222333333333344444444445555555555666666666677777777778888888888999999999900000000001111111111222222222233333333334444444444555555555566666666667777777777888888888899999
        456789012345678901234567890123456789012345678901234567890123456789012345678901234567890123456789012345678901234567890123456789012345678901234567890123456789012345678901234

CRS     TTCTTTCATGGGGAAGCAGATTTGGGTACCACCCAAGTATTGACTCACCCATCAACAACCGCTATGTATTTCGTACATTACTGCCAGCCACCATGAATATTGTACGGTACCATAAATACTTGACCACCTGTAGTACATAAAAACCCAATCCACATCAAAACCCCCTCCCCA

S5 1.1  ............................................................................................................ACCATAAATACTTGACCACCTGTAGTAC
S5 1.1  ............................................................................................................
S5 1.1  ............................................................................................................
S5 1.1  ............................................................................................................
S5 1.1  ............................................................................................................
S5 2.1  ............................................................................................................
S5 2.1  ............................................................................................................
S5 2.1  ............................................................................................................

S5 1.1                                                             CGCTATGTATTTCGTACATTACTGC.......................................................................................
S5 1.1                                                                                      .......................................................................................
S5 1.1                                                                                      .......................................................................................
S5 2.1                                                                                      .................A.....................................................................
S5 2.1                                                                                      .......................................................................................
S5 2.2                                                                                      .......................................................................................
S5 2.2                                                                                      .......................................................................................
S5 2.2                                                                                      .......................................................................................
S5 2.2                                                                                      .......................................................................................

S5 1.1
S5 1.1
S5 1.1
S5 1.2
S5 1.2
S5 2.1
S5 2.1
S5 2.1

S5      ...........................................................................................................................................................................
```

```
1111111111111111111111111111111111111111111111111111111111111111111111111111111111111111111111111111111111111111111111111111111111111111111111111111111111111111111111111111111111111111111111
6666666666666666666666666666666666666666666666666666666666666666666666666666666666666666666666666666666666666666666666666666666666666666666666666666666666666666666666666666666666666666666666
1111122222222222222222222222222222222222222222222222222222222222222222222222222222222222222222222222222222333333333333333333333333333333333333333333333333333333333333333333333333333333333333
9999900000000001111111111222222222233333333334444444444555555555566666666667777777777888888888899999999990000000000111111111122222222223333333333444444444455555555556666666666777777777788888
5678901234567890123456789012345678901234567890123456789012345678901234567890123456789012345678901234567890123456789012345678901234567890123456789012345678901234567890123456789012345678901234

TGCTTACAAGCAAGTACAGCAATCAACCCTCAACTATCACACATCAACTGCAACTCCAAAGCCACCCCTCACCCACTAGGATACCAACAAACCTACCCACCCTTAACAGTACATAGTACATAAAGCCATTTACCGTACATAGCACATTACAGTCAAATCCCTTCTCGTCCCCATGGATGACCCCCCTCAG

..................................................................CCTCACCCACTAGGATACCA
...................................................................
...................................................................
...................................................................
...................................................................
...................................................................
...................................................................
...................................................................
...................................................................

                              CAACTATCACACATCAACTGCAA..............................................T.........C................................................................................
                                                     ..............................................T.........C................................................................................
                                                     ..............................................T.........C................................................................................
                                                     ..............................................T.........C................................................................................
                                                     ..............................................T.........C................................................................................
                                                     ..............................................T.........C................................................................................
                                                     ..............................................T.........C................................................................................
                                                     ..............................................T.........C................................................................................

...................................................................................................T.........C................................................................................

1111111111111111111111111111111111111111111111111111111111111111111111111111111111111111111111111111111111111111111111111111111111111111111111111111111111111111111111111111111111111111111111
6666666666666666666666666666666666666666666666666666666666666666666666666666666666666666666666666666666666666666666666666666666666666666666666666666666666666666666666666666666666666666666666
1111122222222222222222222222222222222222222222222222222222222222222222222222222222222222222222222222222222333333333333333333333333333333333333333333333333333333333333333333333333333333333333
9999900000000001111111111222222222233333333334444444444555555555566666666667777777777888888888899999999990000000000111111111122222222223333333333444444444455555555556666666666777777777788888
5678901234567890123456789012345678901234567890123456789012345678901234567890123456789012345678901234567890123456789012345678901234567890123456789012345678901234567890123456789012345678901234

TGCTTACAAGCAAGTACAGCAATCAACCCTCAACTATCACACATCAACTGCAACTCCAAAGCCACCCCTCACCCACTAGGATACCAACAAACCTACCCACCCTTAACAGTACATAGTACATAAAGCCATTTACCGTACATAGCACATTACAGTCAAATCCCTTCTCGTCCCCATGGATGACCCCCCTCAG

..................................................................CCTCACCCACTAGGATACCA
..................................................................
..................................................................
..................................................................
...C..............................................................
..................................................................
..................................................................
..................................................................
..................................................................

                              CAACTATCACACATCAACTGCAA........................................................C................................................................................
                                                     ........................................................C................................................................................
                                                     ........................................................C................................................................................
                                                     ........................................................C................................................................................
                                                     ........................................................C................................................................................
                                                     ........................................................C................................................................................
                                                     ........................................................C................................................................................
                                                     ........................................................C................................................................................

.............................................................................................................C................................................................................
```

```
         111111111111111111111111111111111111111111111111111111111111111111111111111111111111111111111111111111111111111111111111111111111111111111111111111111111111111111111111111
         666666666666666666666666666666666666666666666666666666666666666666666666666666666666666666666666666666666666666666666666666666666666666666666666666666666666666666666666666
         000000000000000000000000000000000000000000000000000000000000000000000000000011111111111111111111111111111111111111111111111111111111111111111111111111111111111111111111111
         222222333333333344444444445555555555666666666677777777778888888888999999999900000000001111111111222222222233333333334444444444555555555566666666667777777777888888888899999
         456789012345678901234567890123456789012345678901234567890123456789012345678901234567890123456789012345678901234567890123456789012345678901234567890123456789012345678901234

CRS      TTCTTTCATGGGGAAGCAGATTTGGGTACCACCCAAGTATTGACTCACCCATCAACAACCGCTATGTATTTCGTACATTACTGCCAGCCACCATGAATATTGTACGGTACCATAAATACTTGACCACCTGTAGTACATAAAAACCCAATCCACATCAAAACCCCCTCCCCA

S6  1.1  ..........................................................................................A.................ACCATAAATACTTGACCACCTGTAGTAC
S6  1.1  ..........................................................................................A.................
S6  1.1  ..........................................................................................A.................
S6  1.1  .......................................C..................................................A.................
S6  2.1  ..........................................................................................A.................
S6  2.1  ..........................................................................................A.................
S6  2.1  ..........................................................................................A.................
S6  2.1  ..........................................................................................A.................

S6  1.1                                                             CGCTATGTATTTCGTACATTACTGC......A.............................................................................T..
S6  1.1                                                                                      ......A.............................................................................T..
S6  1.1                                                                                      ......A.............................................................................T..
S6  1.2                                                                                      ......A.............................................................................T..
S6  1.2                                                                                      ......A.............................................................................T..
S6  2.1                                                                                      ......A.............................................................................T..
S6  2.1                                                                                      ......A.............................................................................T..
S6  2.1                                                                                      ......A.............................................................................T..
S6  2.1                                                                                      ......A.............................................................................T..

S6  1.1
S6  1.1
S6  1.1
S6  2.1
S6  2.1
S6  2.1
S6  2.1
S6  2.1

S6       ..........................................................................................A.............................................................................T..

         111111111111111111111111111111111111111111111111111111111111111111111111111111111111111111111111111111111111111111111111111111111111111111111111111111111111111111111111111
         666666666666666666666666666666666666666666666666666666666666666666666666666666666666666666666666666666666666666666666666666666666666666666666666666666666666666666666666666
         000000000000000000000000000000000000000000000000000000000000000000000000000011111111111111111111111111111111111111111111111111111111111111111111111111111111111111111111111
         222222333333333344444444445555555555666666666677777777778888888888999999999900000000001111111111222222222233333333334444444444555555555566666666667777777777888888888899999
         456789012345678901234567890123456789012345678901234567890123456789012345678901234567890123456789012345678901234567890123456789012345678901234567890123456789012345678901234

CRS      TTCTTTCATGGGGAAGCAGATTTGGGTACCACCCAAGTATTGACTCACCCATCAACAACCGCTATGTATTTCGTACATTACTGCCAGCCACCATGAATATTGTACGGTACCATAAATACTTGACCACCTGTAGTACATAAAAACCCAATCCACATCAAAACCCCCTCCCCA

S8  1.1  ...............................T............................................................................ACCATAAATACTTGACCACCTGTAGTAC
S8  1.1  ............................................................................................................
S8  1.1  ............................................................................................................
S8  1.1  ............................................................................................................
S8  2.1  ............................................................................................................
S8  2.1  ............................................................................................................
S8  2.1  ............................................................................................................
S8  2.1  ............................................................................................................

S8  1.1                                                             CGCTATGTATTTCGTACATTACTGC.......................................................................................
S8  1.1                                                                                      .......................................................................................
S8  1.1                                                                                      .......................................................................................
S8  1.1                                                                                      .......................................................................................
S8  1.1                                                                                      ............................................................................T..........
S8  2.1                                                                                      .......................................................................................
S8  2.1                                                                                      .......................................................................................
S8  2.1                                                                                      .......................................................................................
S8  2.1                                                                                      .......................................................................................

S8  1.1
S8  1.1
S8  1.1
S8  1.2
S8  1.2
S8  2.1
S8  2.1
S8  2.1

S8       ...........................................................................................................................................................................
```

```
1111111111111111111111111111111111111111111111111111111111111111111111111111111111111111111111111111111111111111111111111111111111111111111111111111111111111111111111111111111111111111111111
6666666666666666666666666666666666666666666666666666666666666666666666666666666666666666666666666666666666666666666666666666666666666666666666666666666666666666666666666666666666666666666666
1111122222222222222222222222222222222222222222222222222222222222222222222222222222222222222222222222222222333333333333333333333333333333333333333333333333333333333333333333333333333333333333
9999900000000001111111111222222222233333333334444444444555555555566666666667777777777888888888899999999990000000000111111111122222222223333333333444444444455555555556666666666777777777788888
5678901234567890123456789012345678901234567890123456789012345678901234567890123456789012345678901234567890123456789012345678901234567890123456789012345678901234567890123456789012345678901234

TGCTTACAAGCAAGTACAGCAATCAACCCTCAACTATCACACATCAACTGCAACTCCAAAGCCACCCCTCACCCACTAGGATACCAACAAACCTACCCACCCTTAACAGTACATAGTACATAAAGCCATTTACCGTACATAGCACATTACAGTCAAATCCCTTCTCGTCCCCATGGATGACCCCCCTCAG

.............................................................T....CCTCACCCACTAGGATACCA
.............................................................T....
.............................................................T....
.............................................................T....
.............................................................T....
.............................................................T....
.............................................................T....
.............................................................T....
.............................................................T....

                              CAACTATCACACATCAACTGCAA........T.....................................T.........C................................................................................
                                                     ........T.....................................T.........C................................................................................
                                                     ........T.....................................T.........C.................................................................................
                                                     ........T.....................................T.........C................................................................................
                                                     ........T.....................................T.........C................................................................................
                                                     ........T.....................................T.........C................................................................................
                                                     ........T.....................................T.........C................................................................................
                                                     ........T.....................................T.........C................................................................................

.............................................................T.....................................T.........C................................................................................

1111111111111111111111111111111111111111111111111111111111111111111111111111111111111111111111111111111111111111111111111111111111111111111111111111111111111111111111111111111111111111111111
6666666666666666666666666666666666666666666666666666666666666666666666666666666666666666666666666666666666666666666666666666666666666666666666666666666666666666666666666666666666666666666666
1111122222222222222222222222222222222222222222222222222222222222222222222222222222222222222222222222222222333333333333333333333333333333333333333333333333333333333333333333333333333333333333
9999900000000001111111111222222222233333333334444444444555555555566666666667777777777888888888899999999990000000000111111111122222222223333333333444444444455555555556666666666777777777788888
5678901234567890123456789012345678901234567890123456789012345678901234567890123456789012345678901234567890123456789012345678901234567890123456789012345678901234567890123456789012345678901234

TGCTTACAAGCAAGTACAGCAATCAACCCTCAACTATCACACATCAACTGCAACTCCAAAGCCACCCCTCACCCACTAGGATACCAACAAACCTACCCACCCTTAACAGTACATAGTACATAAAGCCATTTACCGTACATAGCACATTACAGTCAAATCCCTTCTCGTCCCCATGGATGACCCCCCTCAG

..................................................................CCTCACCCACTAGGATACCA
..................................................................
..................................................................
.................................................A................
..................................................................
..................................................................
..................................................................
..................................................................
..................................................................

                              CAACTATCACACATCAACTGCAA.........................................................................................................................................
                                                     .........................................................................................................................................
                                                     .........................................................................................................................................
                                                     .........................................................................................................................................
                                                     .........................................................................................................................................
                                                     .........................................................................................................................................
                                                     .........................................................................................................................................
                                                     .........................................................................................................................................

..............................................................................................................................................................................................
```

```
            11111111111111111111111111111111111111111111111111111111111111111111111111111111111111111111111111111111111111111111111111111111111111111111111111111111111111111111111111
            66666666666666666666666666666666666666666666666666666666666666666666666666666666666666666666666666666666666666666666666666666666666666666666666666666666666666666666666666
            00000000000000000000000000000000000000000000000000000000000000000000000000001111111111111111111111111111111111111111111111111111111111111111111111111111111111111111111111
            22222233333333334444444444555555555566666666667777777777888888888899999999990000000000111111111122222222223333333333444444444455555555556666666666777777777788888888889999
            45678901234567890123456789012345678901234567890123456789012345678901234567890123456789012345678901234567890123456789012345678901234567890123456789012345678901234567890123

CRS         TTCTTTCATGGGGAAGCAGATTTGGGTACCACCCAAGTATTGACTCACCCATCAACAACCGCTATGTATTTCGTACATTACTGCCAGCCACCATGAATATTGTACGGTACCATAAATACTTGACCACCTGTAGTACATAAAAACCCAATCCACATCAAAACCCCCTCCCC

S9    1.1   ............................................................................................................ACCATAAATACTTGACCACCTGTAGTAC
S9    1.1   ............................................................................................................
S9    1.1   ............................................................................................................
S9    1.1   ............................................................................................................
S9    1.2   ............................................................................................................
S9    1.2   ............................................................................................................
S9    2.1   ............................................................................................................
S9    2.1   ............................................................................................................

S9    1.1                                                              CGCTATGTATTTCGTACATTACTGC......................................................................................
S9    1.1                                                                                       ......................................................................................
S9    1.1                                                                                       ......................................................................................
S9    1.1                                                                                       ........................................................................G.............
S9    2.1                                                                                       ......................................................................................
S9    2.1                                                                                       ......................................................................................
S9    2.1                                                                                       ......................................................................................
S9    2.2                                                                                       ......................................................................................
S9    2.2                                                                                       ......................................................................................

S9    1.1
S9    1.1
S9    1.1
S9    2.1
S9    2.1
S9    2.1
S9    2.1
S9    2.1

S9          ..........................................................................................................................................................................

            11111111111111111111111111111111111111111111111111111111111111111111111111111111111111111111111111111111111111111111111111111111111111111111111111111111111111111111111111
            66666666666666666666666666666666666666666666666666666666666666666666666666666666666666666666666666666666666666666666666666666666666666666666666666666666666666666666666666
            00000000000000000000000000000000000000000000000000000000000000000000000000001111111111111111111111111111111111111111111111111111111111111111111111111111111111111111111111
            22222233333333334444444444555555555566666666667777777777888888888899999999990000000000111111111122222222223333333333444444444455555555556666666666777777777788888888889999
            45678901234567890123456789012345678901234567890123456789012345678901234567890123456789012345678901234567890123456789012345678901234567890123456789012345678901234567890123

CRS         TTCTTTCATGGGGAAGCAGATTTGGGTACCACCCAAGTATTGACTCACCCATCAACAACCGCTATGTATTTCGTACATTACTGCCAGCCACCATGAATATTGTACGGTACCATAAATACTTGACCACCTGTAGTACATAAAAACCCAATCCACATCAAAACCCCCTCCCC

S10   1.1   ............................................................................................................ACCATAAATACTTGACCACCTGTAGTAC
S10   1.1   ............................................................................................................
S10   1.1   ............................................................................................................
S10   1.1   ............................................................................................................
S10   1.2   ...........................................................................................T................
S10   1.2   ............................................................................................................
S10   2.1   ............................................................................................................
S10   2.1   ............................................................................................................

S10   1.1                                                              CGCTATGTATTTCGTACATTACTGC......................................................................................
S10   1.1                                                                                       ......................................................................................
S10   1.1                                                                                       ......................................................................................
S10   1.1                                                                                       ......................................................................................
S10   2.1                                                                                       ......................................................................................
S10   2.1                                                                                       ......................................................................................
S10   2.1                                                                                       ......................................................................................
S10   2.2                                                                                       ......................................................................................
S10   2.2                                                                                       ......................................................................................

S10   1.1
S10   1.1
S10   1.1
S10   2.1
S10   2.1
S10   2.1
S10   2.1
S10   2.1

S10         ..........................................................................................................................................................................
```

```
11111111111111111111111111111111111111111111111111111111111111111111111111111111111111111111111111111111111111111111111111111111111111111111111111111111111111111111111111111111111111111111111
66666666666666666666666666666666666666666666666666666666666666666666666666666666666666666666666666666666666666666666666666666666666666666666666666666666666666666666666666666666666666666666666
11111122222222222222222222222222222222222222222222222222222222222222222222222222222222222222222222222222223333333333333333333333333333333333333333333333333333333333333333333333333333333333333
99999900000000001111111111222222222233333333334444444444555555555566666666667777777777888888888899999999990000000000111111111122222222223333333333444444444455555555556666666666777777777788888
45678901234567890123456789012345678901234567890123456789012345678901234567890123456789012345678901234567890123456789012345678901234567890123456789012345678901234567890123456789012345678901234

ATGCTTACAAGCAAGTACAGCAATCAACCCTCAACTATCACACATCAACTGCAACTCCAAAGCCACCCCTCACCCACTAGGATACCAACAAACCTACCCACCCTTAACAGTACATAGTACATAAAGCCATTTACCGTACATAGCACATTACAGTCAAATCCCTTCTCGTCCCCATGGATGACCCCCCTCAG

...................................................................CCTCACCCACTAGGATACCA
...................................................................
...................................................................
...................................................................
...................................................................
...................................................................
...................................................................
...................................................................
...................................................................

                               CAACTATCACACATCAACTGCAA.........................................................................................................................................
                                                      .........................................................................................................................................
                                                      .........................................................................................................................................
                                                      ......................................................................................................A..................................
                                                      .........................................................................................................................................
                                                      .........................................................................................................................................
                                                      .........................................................................................................................................
                                                      .........................................................................................................................................

...............................................................................................................................................................................................
```


```
11111111111111111111111111111111111111111111111111111111111111111111111111111111111111111111111111111111111111111111111111111111111111111111111111111111111111111111111111111111111111111111111
66666666666666666666666666666666666666666666666666666666666666666666666666666666666666666666666666666666666666666666666666666666666666666666666666666666666666666666666666666666666666666666666
11111122222222222222222222222222222222222222222222222222222222222222222222222222222222222222222222222222223333333333333333333333333333333333333333333333333333333333333333333333333333333333333
99999900000000001111111111222222222233333333334444444444555555555566666666667777777777888888888899999999990000000000111111111122222222223333333333444444444455555555556666666666777777777788888
45678901234567890123456789012345678901234567890123456789012345678901234567890123456789012345678901234567890123456789012345678901234567890123456789012345678901234567890123456789012345678901234

ATGCTTACAAGCAAGTACAGCAATCAACCCTCAACTATCACACATCAACTGCAACTCCAAAGCCACCCCTCACCCACTAGGATACCAACAAACCTACCCACCCTTAACAGTACATAGTACATAAAGCCATTTACCGTACATAGCACATTACAGTCAAATCCCTTCTCGTCCCCATGGATGACCCCCCTCAG

...................................................................CCTCACCCACTAGGATACCA
...................................................................
...................................................................
...................................................................
...................................................................
...................................................................
...................................................................
...................................................................
...................................................................

                               CAACTATCACACATCAACTGCAA.........................................................................................................................................
                                                      .........................................................................................................................................
                                                      .........................................................................................................................................
                                                      ......................................................................................................A..................................
                                                      .........................................................................................................................................
                                                      .........................................................................................................................................
                                                      .........................................................................................................................................
                                                      .........................................................................................................................................

...............................................................................................................................................................................................
```

```
              1111111111111111111111111111111111111111111111111111111111111111111111111111111111111111111111111111111111111111111111111111111111111111111111111111111111111111111111111
              6666666666666666666666666666666666666666666666666666666666666666666666666666666666666666666666666666666666666666666666666666666666666666666666666666666666666666666666666
              0000000000000000000000000000000000000000000000000000000000000000000000000000111111111111111111111111111111111111111111111111111111111111111111111111111111111111111111111
              2222223333333333444444444455555555556666666666777777777788888888889999999999000000000011111111112222222222333333333344444444445555555555666666666677777777778888888888999
              4567890123456789012345678901234567890123456789012345678901234567890123456789012345678901234567890123456789012345678901234567890123456789012345678901234567890123456789012

CRS           TTCTTTCATGGGGAAGCAGATTTGGGTACCACCCAAGTATTGACTCACCCATCAACAACCGCTATGTATTTCGTACATTACTGCCAGCCACCATGAATATTGTACGGTACCATAAATACTTGACCACCTGTAGTACATAAAAACCCAATCCACATCAAAACCCCCTCCC

S11  1.1     ............................................................................................................ACCATAAATACTTGACCACCTGTAGTAC
S11  1.1     ............................................................................................................
S11  1.1     ............................................................................................................
S11  1.1     ............................................................................................................
S11  2.1     .................................................................................................C..........
S11  2.1     ............................................................................................................
S11  2.1     ............................................................................................................
S11  2.1     ............................................................................................................

S11  1.1                                                                          CGCTATGTATTTCGTACATTACTGC....................................................................................T
S11  1.1                                                                                                   ....................................................................................T
S11  1.1                                                                                                   ....................................................................................T
S11  2.1                                                                                                   ....................................................................................T
S11  2.1                                                                                                   ....................................................................................T
S11  2.1                                                                                                   ....................................................................................T
S11  2.1                                                                                                   ....................................................................................T
S11  2.1                                                                                                   ....................................................................................T
S11  2.1                                                                                                   ....................................................................................T

S1 1  1.1
S11  1.1
S11  1.1
S11  1.1
S11  2.1
S11  2.1
S11  2.2
S11  2.2

S11           ........................................................................................................................................................................T

              1111111111111111111111111111111111111111111111111111111111111111111111111111111111111111111111111111111111111111111111111111111111111111111111111111111111111111111111111
              6666666666666666666666666666666666666666666666666666666666666666666666666666666666666666666666666666666666666666666666666666666666666666666666666666666666666666666666666
              0000000000000000000000000000000000000000000000000000000000000000000000000000111111111111111111111111111111111111111111111111111111111111111111111111111111111111111111111
              2222223333333333444444444455555555556666666666777777777788888888889999999999000000000011111111112222222222333333333344444444445555555555666666666677777777778888888888999
              4567890123456789012345678901234567890123456789012345678901234567890123456789012345678901234567890123456789012345678901234567890123456789012345678901234567890123456789012

CRS           TTCTTTCATGGGGAAGCAGATTTGGGTACCACCCAAGTATTGACTCACCCATCAACAACCGCTATGTATTTCGTACATTACTGCCAGCCACCATGAATATTGTACGGTACCATAAATACTTGACCACCTGTAGTACATAAAAACCCAATCCACATCAAAACCCCCTCCC

S17  1.1     ............................................................................................................ACCATAAATACTTGACCACCTGTAGTAC
S17  1.1     ............................................................................................................
S17  1.1     ............................................................................................................
S17  2.1     ............................................................................................................
S17  2.1     ............................................................................................................
S17  2.1     ............................................................................................................
S17  2.1     ............................................................................................................
S17  2.1     ............................................................................................................

S17  1.1                                                                          CGCTATGTATTTCGTACATTACTGC.....................................................................................
S17  1.1                                                                                                   .....................................................................................
S17  1.1                                                                                                   .....................................................................................
S17  1.2                                                                                                   .....................................................................................
S17  1.2                                                                                                   .........CA..........................................................................
S17  1.2                                                                                                   .....................................................................................
S17  1.2                                                                                                   .....................................................................................
S17  2.1                                                                                                   .....................................................................................
S17  2.1                                                                                                   .....................................................................................

S17  1.1
S17  1.1
S17  1.1
S17  1.1
S17  2.1
S17  2.1
S17  2.2
S17  2.2

S17           .........................................................................................................................................................................
```

```
111111111111111111111111111111111111111111111111111111111111111111111111111111111111111111111111111111111111111111111111111111111111111111111111111111111111111111111111111111111111111111111111
666666666666666666666666666666666666666666666666666666666666666666666666666666666666666666666666666666666666666666666666666666666666666666666666666666666666666666666666666666666666666666666666
111111122222222222222222222222222222222222222222222222222222222222222222222222222222222222222222222222222222233333333333333333333333333333333333333333333333333333333333333333333333333333333333
999999900000000001111111111222222222233333333334444444444555555555566666666667777777777888888888899999999990000000000111111111122222222223333333333444444444455555555556666666666777777777788888
345678901234567890123456789012345678901234567890123456789012345678901234567890123456789012345678901234567890123456789012345678901234567890123456789012345678901234567890123456789012345678901234

CATGCTTACAAGCAAGTACAGCAATCAACCCTCAACTATCACACATCAACTGCAACTCCAAAGCCACCCCTCACCCACTAGGATACCAACAAACCTACCCACCCTTAACAGTACATAGTACATAAAGCCATTTACCGTACATAGCACATTACAGTCAAATCCCTTCTCGTCCCCATGGATGACCCCCCTCAG

...............................................................T....CCTCACCCACTAGGATACCA
...............................................................T....
...............................................................T....
...............................................................T....
...............................................................T....
...............................................................T....
...............................................................T....
...............................................................T....
...............................................................T....

                                CAACTATCACACATCAACTGCAA........T................................................................................................................................
                                                       ........T................................................................................................................................
                                                       ........T................................................................................................................................
                                                       ........T................................................................................................................................
                                                       ........T..................................................................................T.............................................
                                                       ........T................................................................................................................................
                                                       ........T................................................................................................................................
                                                       ........T................................................................................................................................
...............................................................T................................................................................................................................

111111111111111111111111111111111111111111111111111111111111111111111111111111111111111111111111111111111111111111111111111111111111111111111111111111111111111111111111111111111111111111111111
666666666666666666666666666666666666666666666666666666666666666666666666666666666666666666666666666666666666666666666666666666666666666666666666666666666666666666666666666666666666666666666666
111111122222222222222222222222222222222222222222222222222222222222222222222222222222222222222222222222222222233333333333333333333333333333333333333333333333333333333333333333333333333333333333
999999900000000001111111111222222222233333333334444444444555555555566666666667777777777888888888899999999990000000000111111111122222222223333333333444444444455555555556666666666777777777788888
345678901234567890123456789012345678901234567890123456789012345678901234567890123456789012345678901234567890123456789012345678901234567890123456789012345678901234567890123456789012345678901234

CATGCTTACAAGCAAGTACAGCAATCAACCCTCAACTATCACACATCAACTGCAACTCCAAAGCCACCCCTCACCCACTAGGATACCAACAAACCTACCCACCCTTAACAGTACATAGTACATAAAGCCATTTACCGTACATAGCACATTACAGTCAAATCCCTTCTCGTCCCCATGGATGACCCCCCTCAG

................C...................................................CCTCACCCACTAGGATACCA
................C...................................................
................C...................................................
................C...................................................
................C...................................................
................C...................................................
................C...................................................
................C...................................................
................C...................................................

                                CAACTATCACACATCAACTGCAA.........................................................................................................................................
                                                       .........................................................................................................................................
                                                       .........................................................................................................................................
                                                       .................................................C.......................................................................................
                                                       .........................................................................................................................................
                                                       .........................................................................................................................................
                                                       .........................................................................................................................................
                                                       .........................................................................................................................................
................C...............................................................................................................................................................................
```
