# Supplementary figures and images for: Origins and Evolution of the Etruscans’ mtDNA
Source: PLoS One. 2013 Feb 6;8(2):e55519. doi: 10.1371/journal.pone.0055519 (PMC3566088; doi:10.1371/journal.pone.0055519)

A

## Mapping Results

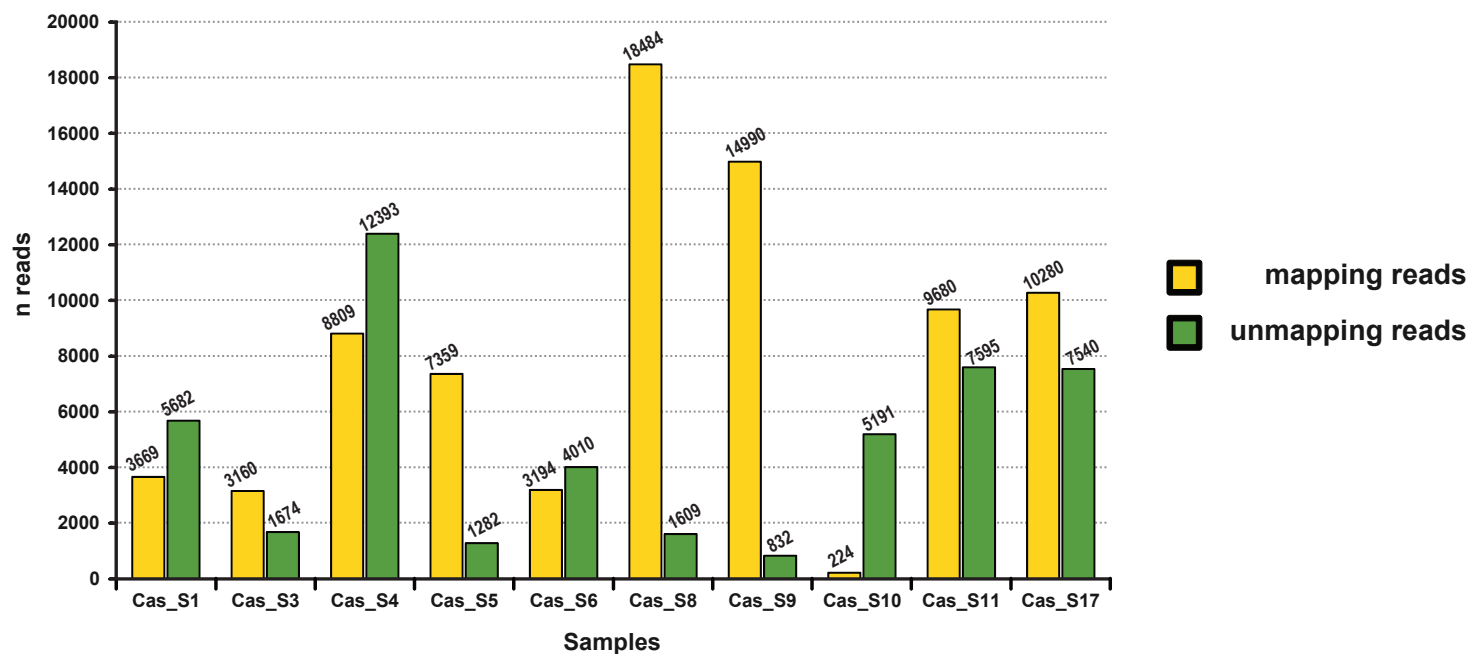

B

## Distribution of the most frequent nucleotide

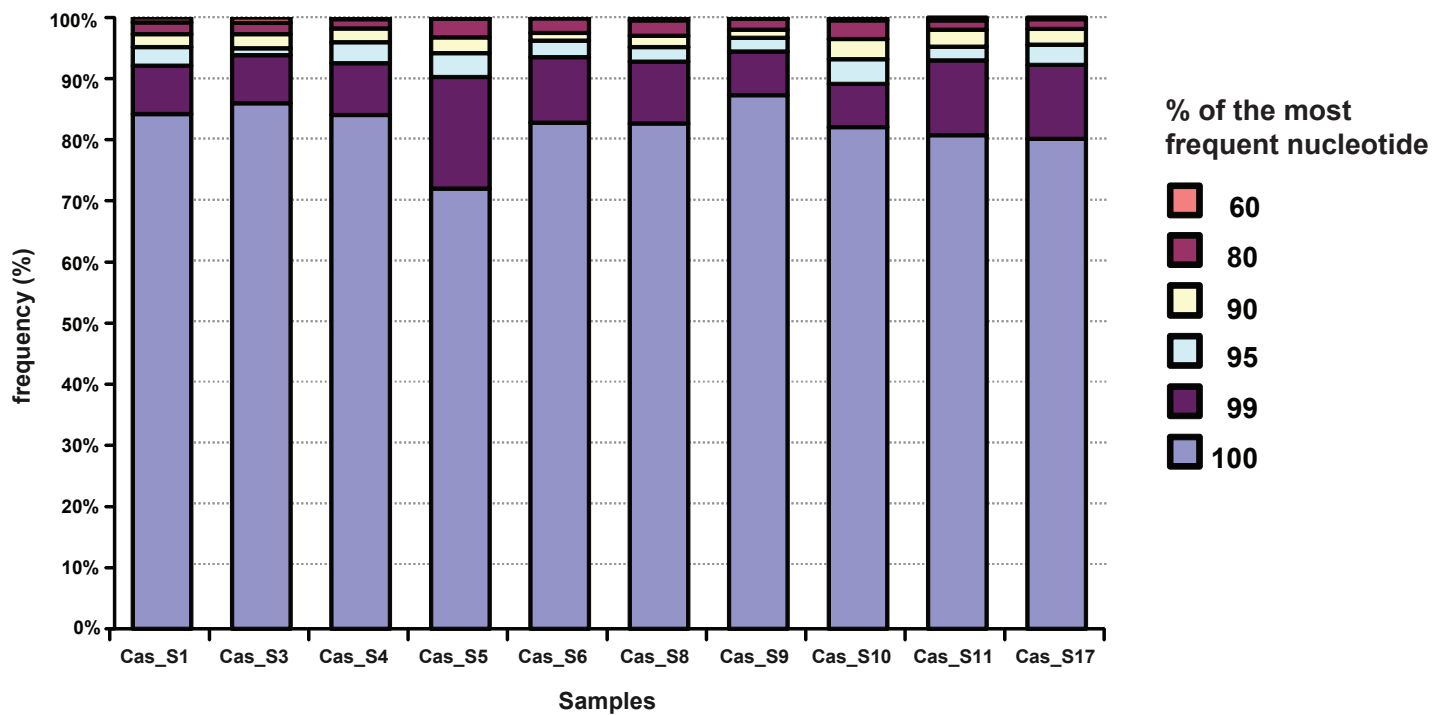

Supplement: Figure S2 — Results of the mapping step for the 10 Etruscan samples analyzed. (A) The number of sequences that map to the reference and those that do not map is plotted as a histogram. Some samples had a large amount of unmapped reads that were afterwards characterized as primers’ dimers. (B) Frequency distribution (% on the Y axis) of the frequency of the most frequent nucleotide for the 10 Etruscan samples analyzed (the upper limits of the % intervals are reported in the legend). For example, in sample S1 at around 84% of the positions the frequency of the most frequent allele among reads is between 99% and 100%. (PDF) [file pone.0055519.s002.pdf]

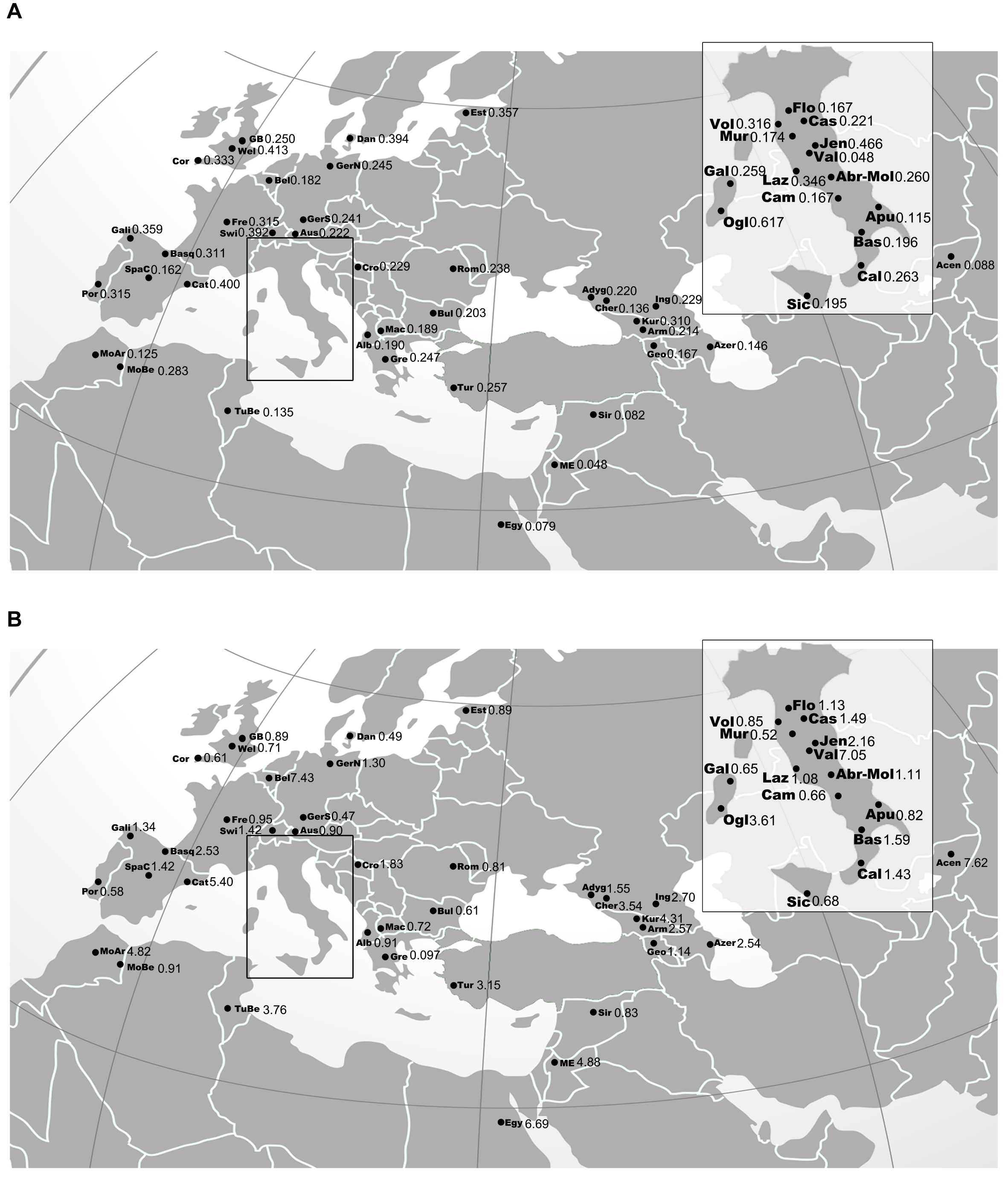

Supplement: Figure S3 — Measures of genetic distance. Allele sharing (A) and Fst (×100) (B) in 52 modern populations of Western Eurasia and the Mediterranean basin. Population labels and sample sizes are provided in Table S2. Allele sharing estimated as the number of sequences shared between Etruscans and every modern population, divided by the sample size of the modern sample. (TIF) [file pone.0055519.s003.tif]

A

## ETR and EUR

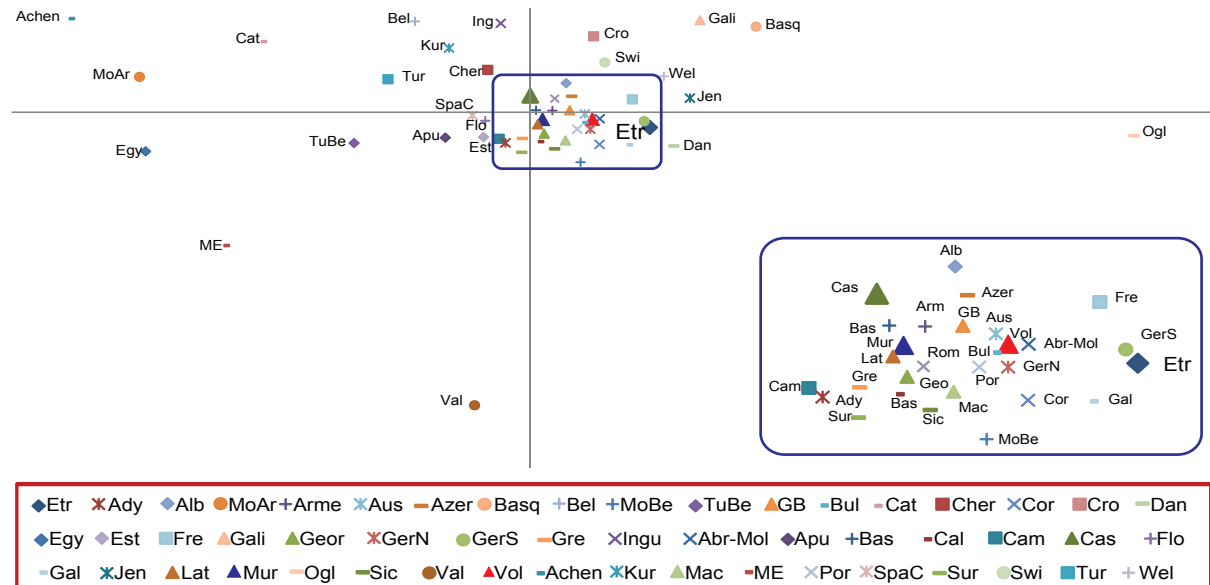

B

## ETR, Medieval and Italian samples

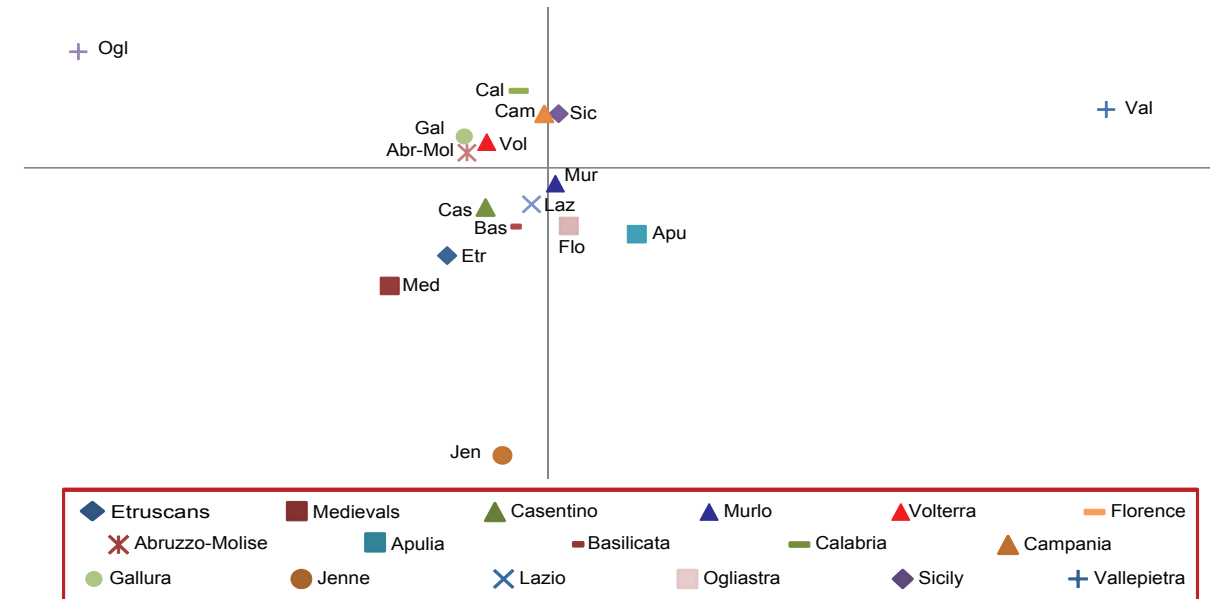

C

## ETR and ANC

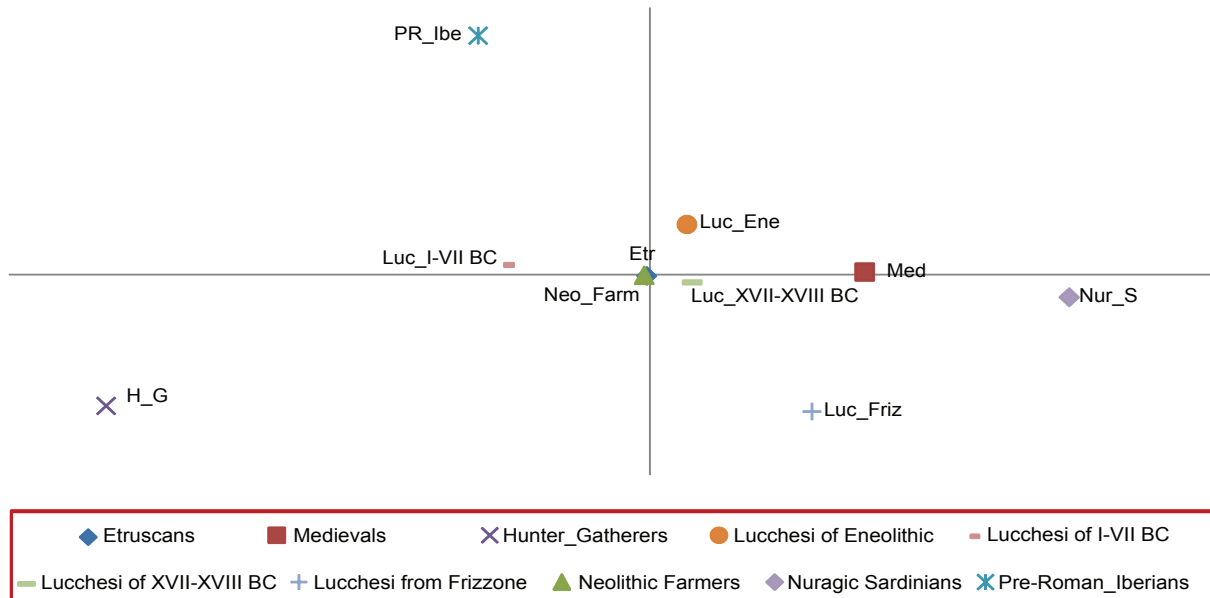

Supplement: Figure S4 — Multi Dimensional Scaling. Multi Dimensional Scaling summarizing genetic affinities between the Etruscans and (A) 52 modern populations of Western Eurasia and the Mediterranean basin; (B) Medieval and modern Italian populations; (C) 9 ancient populations of Europe. Population labels and sample sizes are provided in Table S2. (PDF) [file pone.0055519.s004.pdf]
