# Supplementary material for: Origins and Evolution of the Etruscans’ mtDNA
Source: PLoS One. 2013 Feb 6;8(2):e55519. doi: 10.1371/journal.pone.0055519 (PMC3566088; doi:10.1371/journal.pone.0055519)
Supplement: Figure S5 — Results of model selection. Results of model selection with or without a bottleneck representing the plague epidemics at 625 BP, in Casentino, Murlo and Volterra. Dashed lines represent the presence of plague epidemic that killed one third of the population. For each sample we report the posterior probabilities calculated comparing Models 1–3, either considering or disregarding this demographic event. (PDF) [file pone.0055519.s005.pdf]

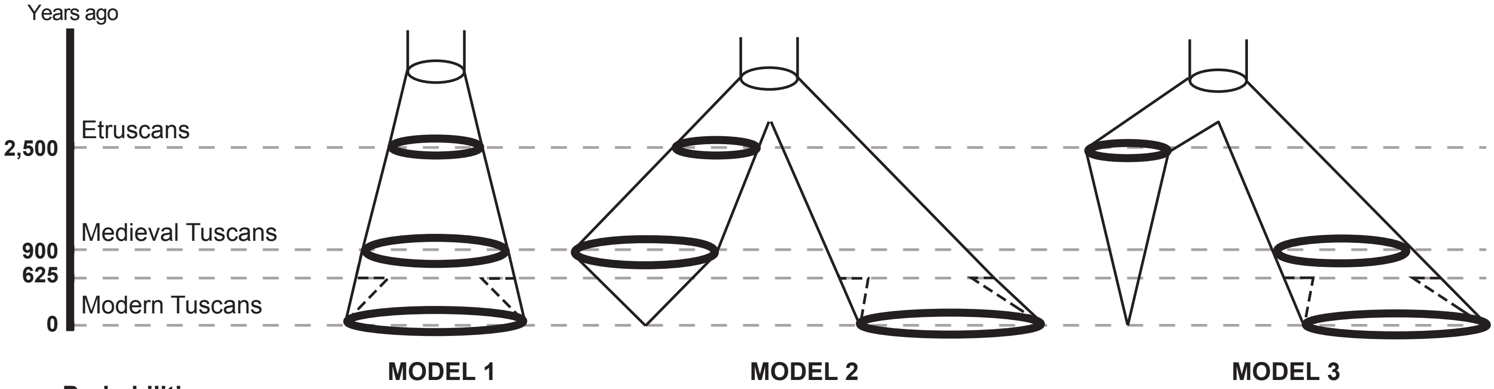

Probabilities

		N	MODEL 1 NoPlague	MODEL 1 Plague	MODEL 2 NoPlague	MODEL 2 Plague	MODEL 3 NoPlague	MODEL 3 Plague
CASENTINO	LR	50,000	**0.986**	**0.998**	0.012	0.000	0.002	0.002
	AR	100	**0.949**	**0.990**	0.040	0.000	0.010	0.000
MURLO	LR	50,000	0.012	0.350	**0.988**	**0.647**	0.000	0.004
	AR	100	0.060	0.360	**0.940**	**0.620**	0.000	0.020
VOLTERRA	LR	50,000	**0.757**	**0.958**	0.227	0.024	0.016	0.017
	AR	100	**0.650**	**0.900**	0.330	0.070	0.020	0.030
