# Supplementary material for: Origins and Evolution of the Etruscans’ mtDNA
Source: PLoS One. 2013 Feb 6;8(2):e55519. doi: 10.1371/journal.pone.0055519 (PMC3566088; doi:10.1371/journal.pone.0055519)

**A**

| | Priors | Median | Mode | 95% HPD-LowB | 95% HPD-UppB | $R^2$ |
|---|---|---|---|---|---|---|
| **Time MRCA** | * | 23,400 | 15,725 | 62,100 | 4,100 | 0.55 |
| **Ne Modern** | (100 - 200,000) | 152,645 | 200,000 | 200,000 | 66,833 | 0.40 |
| **Time Onset Expansion** | (2,550 - 37,500) | 13,575 | 9,175 | 33,450 | 4,125 | 0.28 |
| **Mutation Rate** | (0.0003 - 0.0075) | 0.0027 | 0.0022 | 0.0057 | 0.0009 | 0.64 |
| **Ne Ancestral** | (5 - 6,000) | 216 | 67 | 1,187 | 5 | 0.43 |

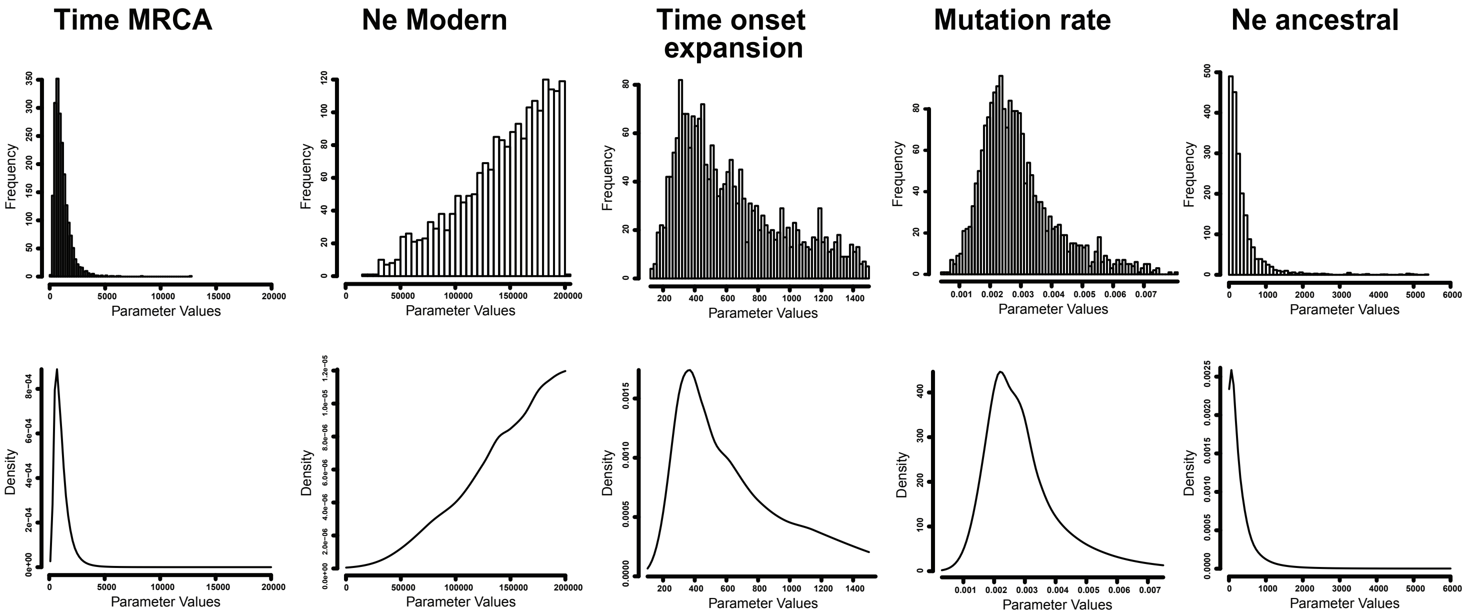


**B**

| | Priors | Median | Mode | 95% HPD-LowB | 95% HPD-UppB | $R^2$ |
|---|---|---|---|---|---|---|
| **Time MRCA** | * | 31,425 | 20,400 | 6,525 | 81,125 | 0.56 |
| **Ne Modern** | (100 - 200,000) | 119,430 | 200,000 | 35,406 | 200,000 | 0.38 |
| **Time Onset Expansion** | (2,550 - 37,500) | 21,625 | 16,450 | 10,300 | 37,500 | 0.28 |
| **Mutation Rate** | (0.0003 - 0.0075) | 0.0023 | 0.0023 | 0.0008 | 0.0048 | 0.64 |
| **Ne Ancestral** | (5 - 6,000) | 388 | 150 | 5 | 1,953 | 0.43 |

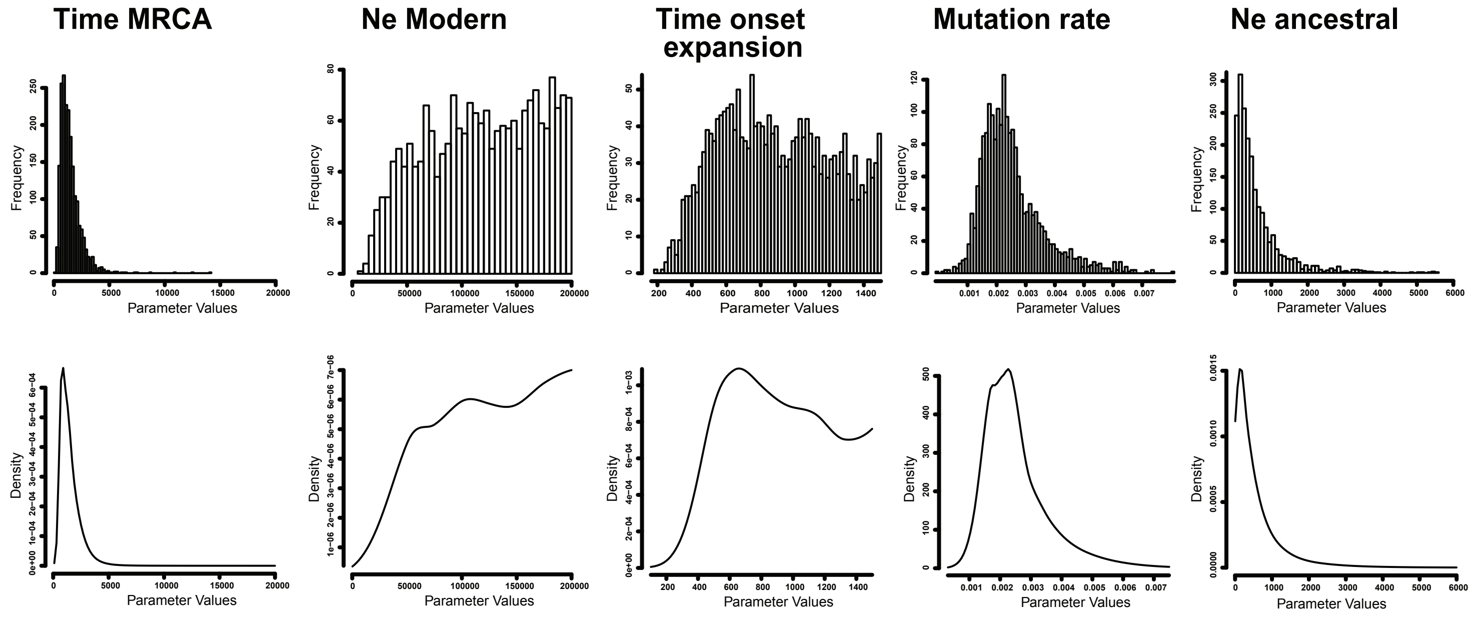

Supplement: Figure S6 — Parameter estimates and posterior distributions under Model 1, for Casentino (A) and Volterra (B). Upper panels: Prior distributions (all the priors were uniform), median and mode estimates, the 95% of the highest posterior density (lower and upper bound), and coefficient of determination R2. The time is expressed in years, the mutation rate in number of mutational events per generation per locus. Lower panels: histograms and smoothed distributions of the parameters estimated. (PDF) [file pone.0055519.s006.pdf]
