# Supplementary material for: Origins and Evolution of the Etruscans’ mtDNA
Source: PLoS One. 2013 Feb 6;8(2):e55519. doi: 10.1371/journal.pone.0055519 (PMC3566088; doi:10.1371/journal.pone.0055519)
Supplement: Figure S7 — IM model (A) and estimates (B) for the separation time between Anatolians and Tuscans. N1 and N2: modern population size; NA: ancestral population size; m1 and m2: migration rates; s: proportion of the ancestral population that founds descendent population 1; t: separation time. Different mutation rates and proportions of the ancestral population founding the descendant populations were considered. (PDF) [file pone.0055519.s007.pdf]

A

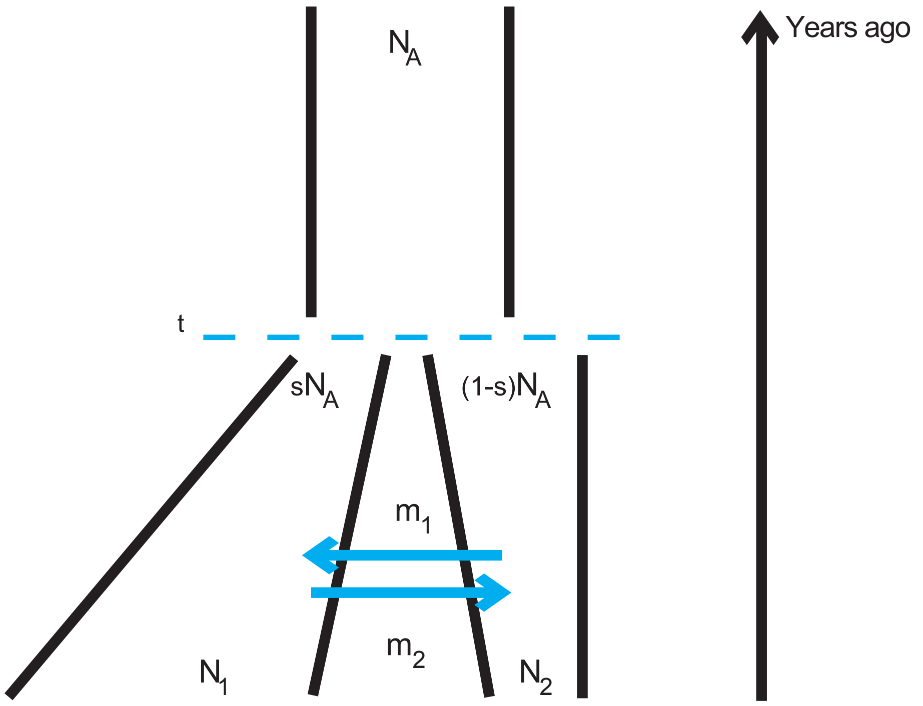

B

Separation Time	Run 1	Run 2	Run3	Run 4
**Mutation Rate = 0.0014**				**s = 0.5**
**Mean**	16,180	16,939	16,861	15,739
**95% LowB CI**	12,500	12,461	12,020	11,780
**95% UppB CI**	20,620	22,020	22,700	21,580
**Mutation Rate = 0.003**				**s = 0.5**
**Mean**	7,775	7,980	7,663	6,692
**95% LowB CI**	5,572	5,945	5,665	6,002
**95% UppB CI**	9,958	10,556	10,444	9,861
**Mutation Rate = 0.006**				**s = 0.5**
**Mean**	3,775	3,915	3,748	3,831
**95% LowB CI**	2,683	2,954	2,749	2,935
**95% UppB CI**	4,989	5,017	4,839	4,905
**Mutation Rate = 0.003**		**s = 0.95**		
**Mean**	9,183	9,146		
**95% LowB CI**	6,698	6,474		
**95% UppB CI**	12,583	13,088		
