# Supplementary material for: Origins and Evolution of the Etruscans’ mtDNA
Source: PLoS One. 2013 Feb 6;8(2):e55519. doi: 10.1371/journal.pone.0055519 (PMC3566088; doi:10.1371/journal.pone.0055519)
Supplement: Table S1 — Consensus HVR-I Etruscans mtDNA and sequences of all the investigators. Upper panel: Consensus HVR-I mtDNA sequences in 30 individuals from historical Etruria. Tarq represents individuals from Tarquinia, Cas from Casenovole, Vol from Volterra, Pie from Castelluccio di Pienza, Sot from Castelfranco di Sotto and MM from Magliano and Marsiliana. CRS is the Cambridge reference sequence [32]. The HVR-I motif is the position (−16,000) where substitution were observed, with respect to the CRS; the observed transversions are indicated with a capital letter. The haplotypes shared with EUR dataset are in bold type. For the Casenovole sample, the labels of the individuals used in Figure S1 are between parentheses. Lower panel: Sequences of all the investigators who had direct contact with the ancient specimens. (DOCX) [file pone.0055519.s008.docx]

| **Sequence label** | **Century (BC)** | **HVR1 motif (16024-16384)** | | **Haplotype** | **Reference** |  |
| --- | --- | --- | --- | --- | --- | --- |
| Tarq_1 | 3rd | 069, 126, 193 | | **Hap16** | This study |  |
| Tarq_2 | 4th-3rd | CRS | | **Hap1** | This study |  |
| Tarq_3 | 6th | 270 | | **Hap17** | This study |  |
| Tarq_4 | 3rd-2nd | CRS | | **Hap1** | This study |  |
| Tarq_5 | 3rd | 126, 229, 362 | | Hap18 | [1] |  |
| Tarq_6 | 5th | 126, 193 | | **Hap15** | [1] |  |
| Tarq_7 | 3rd | 126, 193, 228, 229, 278 | | Hap19 | [1] |  |
| Tarq_8 | 5th | 278, 334 | | Hap20 | [1] |  |
| Tarq_9 | 3rd | 098, 311, 327 | | Hap21 | [1] |  |
| Cas_1 (S1) | 3rd | 192 | | **Hap1** | This study |  |
| Cas_2 (S10) | 3rd | CRS | | **Hap1** | This study |  |
| Cas_3 (S11) | 3rd | 192, 256 | | **Hap6** | This study |  |
| Cas_4 (S17) | 3rd | 209 | | **Hap3** | This study |  |
| Cas_5 (S3) | 3rd | 192, 256 | | **Hap6** | This study |  |
| Cas_6 (S4) | 3rd | 114A, 192,294, 304 | | Hap4 | This study |  |
| Cas_7 (S5) | 3rd | 304 | | **Hap2** | This study |  |
| Cas_8 (S6) | 3rd | 114A, 192, 256, 294, 304 | | Hap5 | This study |  |
| Cas_9 (S8) | 3rd | CRS | | **Hap1** | This study |  |
| Cas_10 (S9) | 3rd | CRS | | **Hap1** | This study |  |
| Vol_1 | 6th-5th | 193, 219 | | **Hap7** | [1] |  |
| Vol_2 | 2nd-1st | 189, 274, 334, 356 | | Hap8 | [1] |  |
| Vol_3 | 6th-5th | 261 | | **Hap9** | [1] |  |
| Pie_1 | ? | 193, 219, 256, 270, 291 | | Hap10 | [1] |  |
| Sot_1 | ? | 189, 356 | | **Hap11** | [1] |  |
| MM_1 | 7th-6th | CRS | | **Hap1** | [1] |  |
| MM_2 | 6th | 126 | | **Hap15** | [1] |  |
| MM_3 | 6th | 126, 193 | | **Hap15** | [1] |  |
| MM_4 | 6th | 095G, 126, 189 | | Hap12 | [1] |  |
| MM_5 | 7th-6th | 066, 126, 193, 219 | | Hap13 | [1] |  |
| MM_6 | 6th | 311 | | **Hap14** | [1] |  |
|  | | | | | |  |
| **Researcher** | **Task** | | **HVR1 haplotype** | | | |
| E.P | Excavation | | 16165 G, 16222 T | | | |
| S.V | Ancient DNA Laboratory analysis | | 16311 C | | | |
| A.S | Ancient DNA Laboratory analysis | | 16145 A | | | |
| M.L | Ancient DNA Laboratory analysis | | 16261 T, 16311 C | | | |
| D.C. | Ancient DNA Laboratory analysis | | 16193 T, 16278 T | | | |

**References**

1. Vernesi C, Caramelli D, Dupanloup I, Bertorelle G, Lari M, et al. (2004) The Etruscans: a population-genetic study. Am J Hum Genet 74: 694-704.
