# Supplementary material for: Origins and Evolution of the Etruscans’ mtDNA
Source: PLoS One. 2013 Feb 6;8(2):e55519. doi: 10.1371/journal.pone.0055519 (PMC3566088; doi:10.1371/journal.pone.0055519)
Supplement: Table S2 — Detailed description of the samples in the EUR and ANC datasets. (DOC) [file pone.0055519.s009.doc]

**Supplementary table 2:** Detailed description of the samples in the EUR and ANC datasets.

| **Population** | **ID** | **Region** | **n** | **Reference** |
| --- | --- | --- | --- | --- |
| **EUR** | | | | |
| Adygei | Ady | Caucasus | 50 | [1] |
| Albanians | Alb | Europe, SouthEast | 84 | [2,3] |
| Arabs, Maroc | MoAr | North Africa | 32 | [4] |
| Armenians | Arme | Caucasus | 42 | [5] |
| Austrians | Aus | Europe, Central | 117 | [6,7] |
| Azerbaijani | Azer | Caucasus | 41 | [5] |
| Basques | Basq | Europe, West | 106 | [8,9] |
| Belgians | Bel | Europe, Central | 33 | [10] |
| Berbers, Maroc | MoBe | North Africa | 60 | [11,4] |
| Berbers, Tunisia | TuBe | North Africa | 155 | [12] |
| British | GB | Europe, North | 100 | [13] |
| Bulgarians | Bul | Europe,SouthEast | 882 | [14,15] |
| Catalans | Cat | Europe, West | 15 | [9] |
| Cherkessians | Cher | Caucasus | 44 | [5] |
| Cornish | Cor | Europe, NorthWest | 69 | [16] |
| Croatians | Cro | Europe, SouthEast | 96 | [17] |
| Danes | Dan | Europe, North | 32 | [16] |
| Egyptians | Egy | North Africa | 124 | [18,19] |
| Estonians | Est | Europe, North | 28 | [20] |
| French | Fre | Europe, Central | 111 | [21] |
| Galicians | Gali | Europe, West | 92 | [22] |
| Georgians | Geo | Caucasus | 102 | [5,23] |
| Germans, North | GerN | Europe, North | 108 | [16] |
| Germans, South | GerS | Europe, Central | 249 | [16,24] |
| Greeks | Gre | Europe, SouthEast | 73 | [3,25] |
| Ingush | Ingu | Caucasus | 35 | [5] |
| Italians, Abruzzo_Molise | Abr-Mol | Italy, Central | 73 | [17] |
| Italians, Apulia | Apu | Italy, South | 26 | [17] |
| Italians, Basilicata | Bas | Italy, South | 92 | [26] |
| Italians, Calabria | Cal | Italy, South | 95 | [26] |
| Italians, Campania | Cam | Italy, South | 48 | [17] |
| Italians, Casentino | Cas | Italy, Central | 122 | [27] |
| Italians, Florence | Flo | Italy, Central | 48 | [28] |
| Italians, Gallura | Gal | Italy,Sardinia | 27 | [29] |
| Italians, Jenne | Jen | Italy, Central | 103 | [30] |
| Italians, Latium | Lat | Italy, Central | 52 | [17] |
| Italians, Murlo | Mur | Italy, Central | 86 | [27] |
| Italians, Ogliastra | Ogl | Italy,Sardinia | 175 | [31] |
| Italians, Sicily | Sic | Italy, South | 154 | [26] |
| Italians, Vallepietra | Val | Italy, Central | 21 | [29] |
| Italians, Volterra | Vol | Italy, Central | 114 | [27] |
| Kazakhs, Kirghizs,Uyghurs | Achen | Central Asia | 205 | [32] |
| Kurds | Kur | Near East | 29 | [32] |
| Macedonians | Mac | Europe, SouthEast | 37 | [3] |
| Middle East | ME | Near East | 42 | [33] |
| Portuguese | Por | Europe, West | 54 | [9] |
| Romanians | Rom | Europe, SouthEast | 105 | [3] |
| Spaniards, Central | SpaC | Europe, West | 74 | [9,11] |
| Surians | Sur | Near East | 49 | [25] |
| Swiss | Swi | Europe, Central | 72 | [34] |
| Turks, Anatolia | Tur | Near East | 35 | [35,36] |
| Welsh | Wel | Europe, North | 92 | [16] |
|  | | | | |
| **ANC** | | | | |
| Medieval Tuscans | Med | Italy, Central | 27 | [37] |
| Neolithic Farmers | Neo_Farm | Europe, Central | 71 | [38,39] |
| Hunter-Gatherers Europeans | H_G | Europe, Central | 20 | [40] |
| Pre-Roman Iberian | PR_Ibe | Europe, West | 17 | [41] |
| Lucchesi of Eneolithic | Luc_Ene | Italy, Central | 10 | unpublished data |
| Lucchesi from Frizzone | Luc_Friz | Italy, Central | 8 | unpublished data |
| Lucchesi of I-VII BC | Luc_ I-VIIBC | Italy, Central | 4 | unpublished data |
| Lucchesi of XVI-XVIII BC | Luc_ XVI-XVIII BC | Italy, Central | 10 | unpublished data |
| Nuragic Sardians | Nur_S | Italy,Sardinia | 23 | [42] |

**References**

1. Macaulay V, Richards M, Hickey E, Vega E, Cruciani F, et al. (1999) The emerging tree of West Eurasian mtDNAs: a synthesis of control-region sequences and RFLPs. Am J Hum Genet 64: 232-249.

2. Belledi M, Poloni ES, Casalotti R, Conterio F, Mikerezi I, et al. (2000) Maternal and paternal lineages in Albania and the genetic structure of Indo-European populations. Eur J Hum Genet 8: 480-486.

3. Bosch E, Calafell F, Gonzalez-Neira A, Flaiz C, Mateu E, et al. (2006) Paternal and maternal lineages in the Balkans show a homogeneous landscape over linguistic barriers, except for the isolated Aromuns. Ann Hum Genet 70: 459-487.

4. Rando JC, Pinto F, Gonzalez AM, Hernandez M, Larruga JM, et al. (1998) Mitochondrial DNA analysis of northwest African populations reveals genetic exchanges with European, near-eastern, and sub-Saharan populations. Ann Hum Genet 62: 531-550.

5. Nasidze I, Stoneking M (2001) Mitochondrial DNA variation and language replacements in the Caucasus. Proc Biol Sci 268: 1197-1206.

6. Handt O, Richards M, Trommsdorff M, Kilger C, Simanainen J, et al. (1994) Molecular genetic analyses of the Tyrolean Ice Man. Science 264: 1775-1778.

7. Parson W, Parsons TJ, Scheithauer R, Holland MM (1998) Population data for 101 Austrian Caucasian mitochondrial DNA d-loop sequences: application of mtDNA sequence analysis to a forensic case. Int J Legal Med 111: 124-132.

8. Bertranpetit J, Sala J, Calafell F, Underhill PA, Moral P, et al. (1995) Human mitochondrial DNA variation and the origin of Basques. Ann Hum Genet 59: 63-81.

9. Corte-Real HB, Macaulay VA, Richards MB, Hariti G, Issad MS, et al. (1996) Genetic diversity in the Iberian Peninsula determined from mitochondrial sequence analysis. Ann Hum Genet 60: 331-350.

10. Decorte R, Jehaes E, Xiao FX, Cassiman J-J (1996) Genetic analysis of single hair shafts by automated sequence analysis of the mitochondrial d-loop region. Advances in Forensic Haemogenetics 6: 17-19.

11. Pinto F, Gonzalez AM, Hernandez M, Larruga JM, Cabrera VM (1996) Genetic relationship between the Canary Islanders and their African and Spanish ancestors inferred from mitochondrial DNA sequences. Ann Hum Genet 60: 321-330.

12. Fadhlaoui-Zid K, Plaza S, Calafell F, Ben Amor M, Comas D, et al. (2004) Mitochondrial DNA heterogeneity in Tunisian Berbers. Ann Hum Genet 68: 222-233.

13. Piercy R, Sullivan KM, Benson N, Gill P (1993) The application of mitochondrial DNA typing to the study of white Caucasian genetic identification. Int J Legal Med 106: 85-90.

14. Calafell F, Underhill P, Tolun A, Angelicheva D, Kalaydjieva L (1996) From Asia to Europe: mitochondrial DNA sequence variability in Bulgarians and Turks. Ann Hum Genet 60: 35-49.

15. Karachanak S, Carossa V, Nesheva D, Olivieri A, Pala M, et al. (2011) Bulgarians vs the other European populations: a mitochondrial DNA perspective. Int J Legal Med.

16. Richards M, Corte-Real H, Forster P, Macaulay V, Wilkinson-Herbots H, et al. (1996) Paleolithic and neolithic lineages in the European mitochondrial gene pool. Am J Hum Genet 59: 185-203.

17. Babalini C, Martinez-Labarga C, Tolk HV, Kivisild T, Giampaolo R, et al. (2005) The population history of the Croatian linguistic minority of Molise (southern Italy): a maternal view. Eur J Hum Genet 13: 902-912.

18. Krings M, Salem AE, Bauer K, Geisert H, Malek AK, et al. (1999) mtDNA analysis of Nile River Valley populations: A genetic corridor or a barrier to migration? Am J Hum Genet 64: 1166-1176.

19. Stevanovitch A, Gilles A, Bouzaid E, Kefi R, Paris F, et al. (2004) Mitochondrial DNA sequence diversity in a sedentary population from Egypt. Ann Hum Genet 68: 23-39.

20. Sajantila A, Lahermo P, Anttinen T, Lukka M, Sistonen P, et al. (1995) Genes and languages in Europe: an analysis of mitochondrial lineages. Genome Res 5: 42-52.

21. Cali F, Le Roux MG, D'Anna R, Flugy A, De Leo G, et al. (2001) MtDNA control region and RFLP data for Sicily and France. Int J Legal Med 114: 229-231.

22. Salas A, Comas D, Lareu MV, Bertranpetit J, Carracedo A (1998) mtDNA analysis of the Galician population: a genetic edge of European variation. Eur J Hum Genet 6: 365-375.

23. Comas D, Calafell F, Bendukidze N, Fananas L, Bertranpetit J (2000) Georgian and kurd mtDNA sequence analysis shows a lack of correlation between languages and female genetic lineages. Am J Phys Anthropol 112: 5-16.

24. Lutz S, Weisser HJ, Heizmann J, Pollak S (1998) Location and frequency of polymorphic positions in the mtDNA control region of individuals from Germany. Int J Legal Med 111: 67-77.

25. Vernesi C, Di Benedetto G, Caramelli D, Secchieri E, Simoni L, et al. (2001) Genetic characterization of the body attributed to the evangelist Luke. Proc Natl Acad Sci U S A 98: 13460-13463.

26. Ottoni C, Martinez-Labarga C, Vitelli L, Scano G, Fabrini E, et al. (2009) Human mitochondrial DNA variation in Southern Italy. Ann Hum Biol 36: 785-811.

27. Achilli A, Olivieri A, Pala M, Metspalu E, Fornarino S, et al. (2007) Mitochondrial DNA variation of modern Tuscans supports the near eastern origin of Etruscans. Am J Hum Genet 80: 759-768.

28. Turchi C, Buscemi L, Previdere C, Grignani P, Brandstatter A, et al. (2008) Italian mitochondrial DNA database: results of a collaborative exercise and proficiency testing. Int J Legal Med 122: 199-204.

29. Morelli L, Grosso MG, Vona G, Varesi L, Torroni A, et al. (2000) Frequency distribution of mitochondrial DNA haplogroups in Corsica and Sardinia. Hum Biol 72: 585-595.

30. Messina F, Scorrano G, Labarga CM, Rolfo MF, Rickards O (2010) Mitochondrial DNA variation in an isolated area of Central Italy. Ann Hum Biol 37: 385-402.

31. Fraumene C, Petretto E, Angius A, Pirastu M (2003) Striking differentiation of sub-populations within a genetically homogeneous isolate (Ogliastra) in Sardinia as revealed by mtDNA analysis. Hum Genet 114: 1-10.

32. Comas D, Calafell F, Mateu E, Perez-Lezaun A, Bosch E, et al. (1998) Trading genes along the silk road: mtDNA sequences and the origin of central Asian populations. Am J Hum Genet 63: 1824-1838.

33. Di Rienzo A, Wilson AC (1991) Branching pattern in the evolutionary tree for human mitochondrial DNA. Proc Natl Acad Sci U S A 88: 1597-1601.

34. Pult I, Sajantila A, Simanainen J, Georgiev O, Schaffner W, et al. (1994) Mitochondrial DNA sequences from Switzerland reveal striking homogeneity of European populations. Biol Chem Hoppe Seyler 375: 837-840.

35. Di Benedetto G, Erguven A, Stenico M, Castri L, Bertorelle G, et al. (2001) DNA diversity and population admixture in Anatolia. Am J Phys Anthropol 115: 144-156.

36. Quintana-Murci L, Chaix R, Wells RS, Behar DM, Sayar H, et al. (2004) Where west meets east: the complex mtDNA landscape of the southwest and Central Asian corridor. Am J Hum Genet 74: 827-845.

37. Guimaraes S, Ghirotto S, Benazzo A, Milani L, Lari M, et al. (2009) Genealogical discontinuities among Etruscan, Medieval, and contemporary Tuscans. Mol Biol Evol 26: 2157-2166.

38. Haak W, Forster P, Bramanti B, Matsumura S, Brandt G, et al. (2005) Ancient DNA from the first European farmers in 7500-year-old Neolithic sites. Science 310: 1016-1018.

39. Lacan M, Keyser C, Ricaut FX, Brucato N, Duranthon F, et al. (2011) Ancient DNA reveals male diffusion through the Neolithic Mediterranean route. Proc Natl Acad Sci U S A 108: 9788-9791.

40. Bramanti B, Thomas MG, Haak W, Unterlaender M, Jores P, et al. (2009) Genetic discontinuity between local hunter-gatherers and central Europe's first farmers. Science 326: 137-140.

41. Sampietro ML, Caramelli D, Lao O, Calafell F, Comas D, et al. (2005) The genetics of the pre-Roman Iberian Peninsula: a mtDNA study of ancient Iberians. Ann Hum Genet 69: 535-548.

42. Caramelli D, Vernesi C, Sanna S, Sampietro L, Lari M, et al. (2007) Genetic variation in prehistoric Sardinia. Hum Genet 122: 327-336.
